# Supplementary figures and images for: Prophage-like gene transfer agents promote Caulobacter crescentus survival and DNA repair during stationary phase
Source: PLoS Biol. 2022 Nov 3;20(11):e3001790. doi: 10.1371/journal.pbio.3001790 (PMC9632790; doi:10.1371/journal.pbio.3001790)

Figure S1

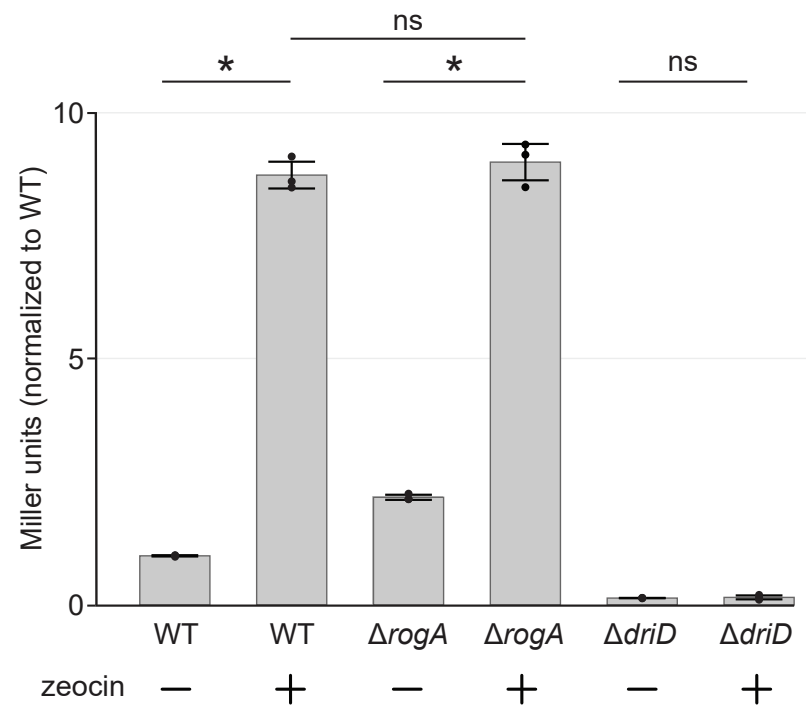

Supplement: S1 Fig — β-galactosidase assay measuring transcriptional activity of a didA reporter in WT, ΔrogA and ΔdriD cells grown up to early stationary phase and treated with or without 15 μg/mL zeocin for 45 min (n = 3, error bars indicate SD). * = p-value <0.05 of indicated comparisons. ns = not significant. Data are available in S1 Data. (PDF) [file pbio.3001790.s001.pdf]

Figure S3

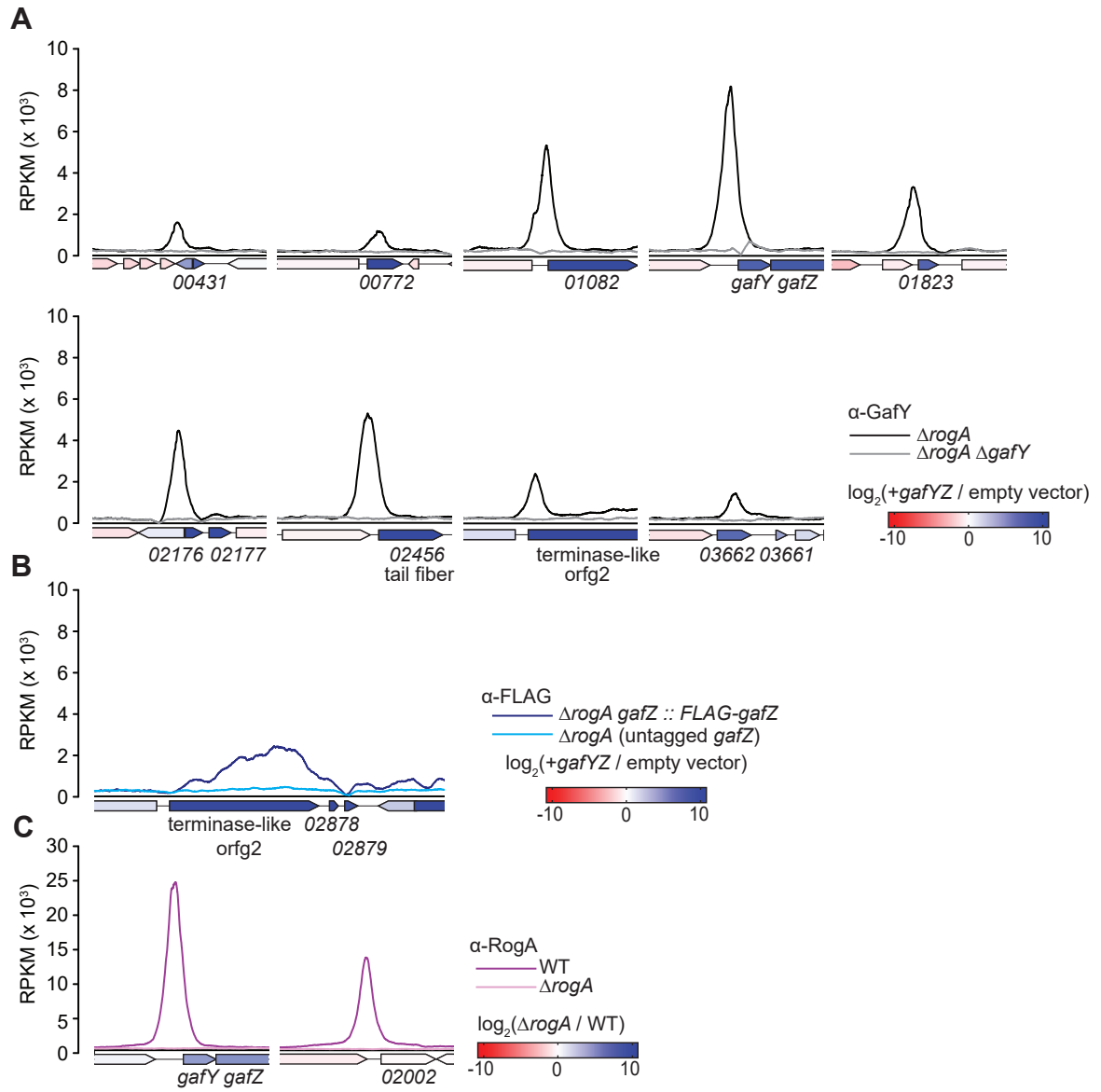

Supplement: S3 Fig — (A) Individual ChIP-seq profiles of GafY with an anti-GafY antibody in either ΔrogA (black) or ΔrogA ΔgafY (gray). Profiles were plotted with the x-axis representing genomic positions and the y-axis representing the number of reads per kilobase pair per million mapped reads (RPKM) using custom R scripts. Corresponding gene annotations are shown with comparative RNA-seq data as shown before. Data are available in S1 Data. (B) ChIP-seq profile of the 1 identified peak of FLAG-GafZ with an anti-FLAG antibody in either ΔrogA gafZ::FLAG-gafZ (dark blue) or ΔrogA (light blue). Profiles were plotted as in S3A Fig. Data are available in S1 Data. (C) Individual ChIP-seq profiles of the 2 identified peaks of RogA with an anti-RogA antibody in either WT (purple) or ΔrogA (light purple). Profiles were plotted as in S3A Fig. Data are available in S1 Data. (PDF) [file pbio.3001790.s003.pdf]

Figure S4

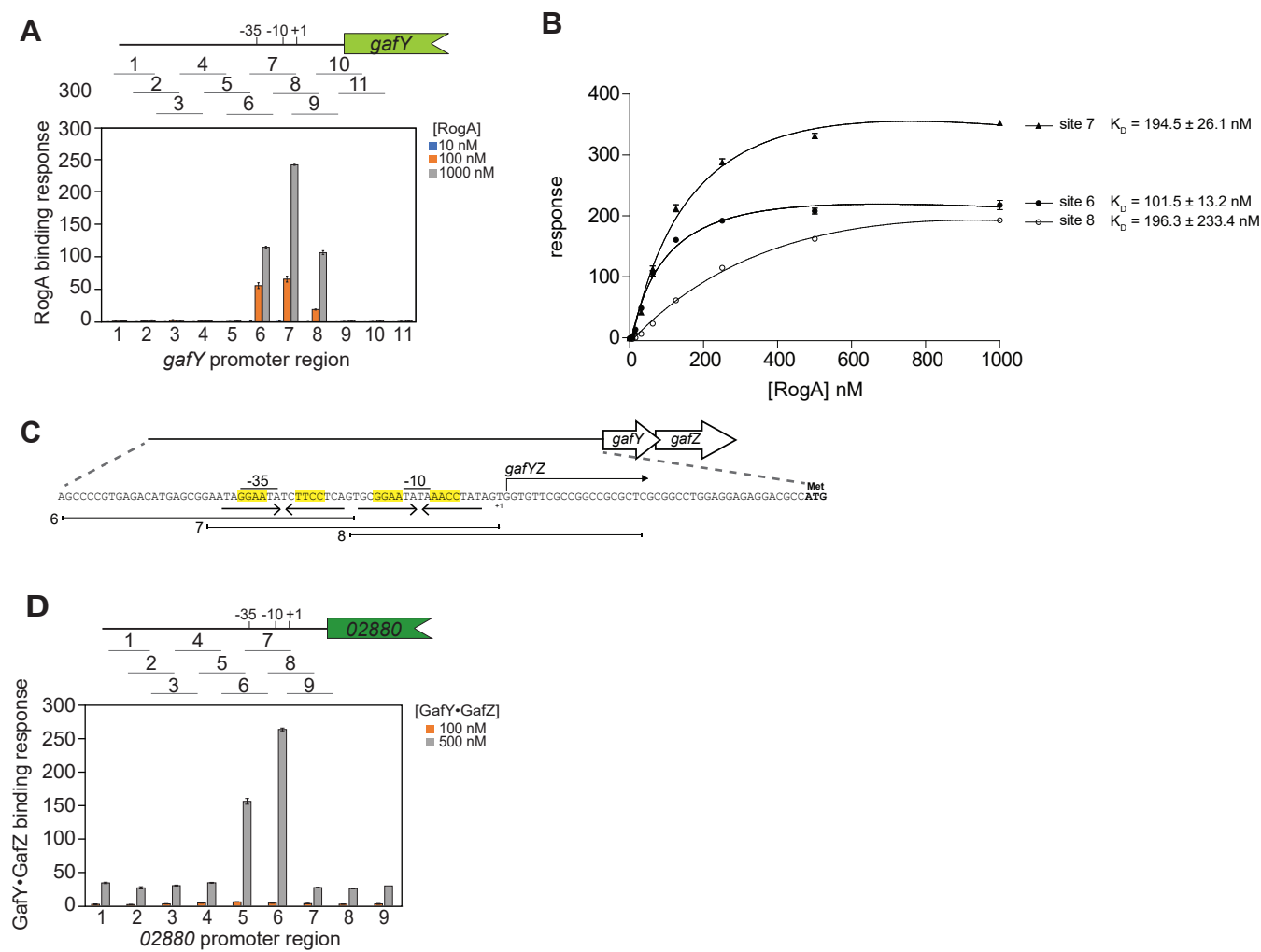

Supplement: S4 Fig — (A) Surface plasmon resonance analysis of purified RogA binding to several overlapping segments of the gafYZ promoter. Data are available in S1 Data. (B) SPR response of RogA-His6 to the 3 best binding probes as a function of protein concentration. Corresponding Kd is shown. Data are available in S1 Data. (C) Probe position relative to the gafYZ promoter with potential inverted repeats highlighted in yellow. (D) SPR response of purified GafY•GafZ complex to various DNA probes corresponding to the GTA cluster promoter. Probe position relative to the promoter is shown above. Data are available in S1 Data. (PDF) [file pbio.3001790.s004.pdf]

Figure S5

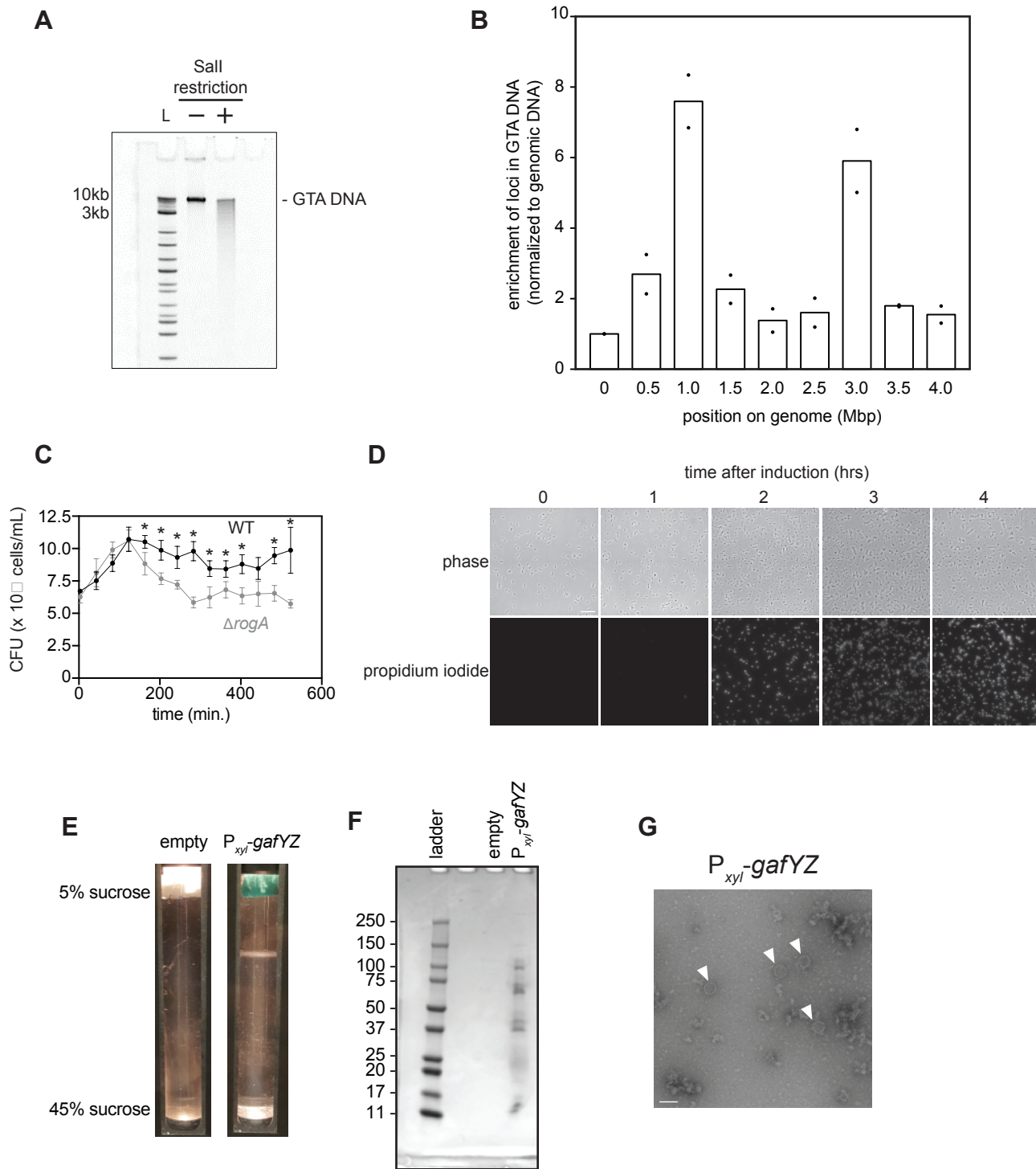

Supplement: S5 Fig — (A) SalI restriction digestion of purified GTA DNA run on a 6% TBE gel. (B) Quantitative PCR of different genomic regions in the GTA DNA. Relative enrichment of each locus is normalized to quantification using genomic DNA as input. Data are available in S1 Data. (C) Time course of quantification of colony-forming units of WT and ΔrogA cells starting at OD 0.75, with time points every 40 min. * = p-value <0.05 comparing data of same time point. Data are available in S1 Data. (D) Propidium iodide (1 μm final concentration) staining of cells overexpressing gafYZ from a high-copy vector (Pxyl-gafYZ) with phase images. Scale bar = 10 μm. (E) Approximately 5% to 45% sucrose gradients of purified GTA samples from cells with a plasmid bearing either Pxyl-empty or Pxyl-gafYZ. (F) SDS-PAGE gel of proteins extracted from GTAs purified with ultracentrifugation through sucrose gradients. (G) Transmission electron micrographs of PEG-precipitated filtered supernatant from cells overexpressing gafYZ from the Pxyl-gafYZ high-copy plasmid in stationary phase. Scale bar = 100 nm. Particles noted with white arrows. (PDF) [file pbio.3001790.s005.pdf]

**A**

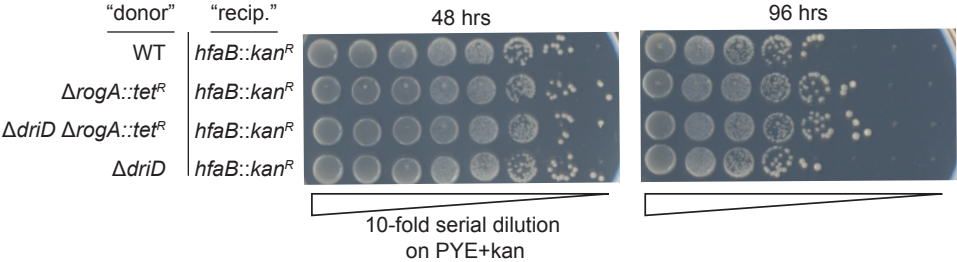

**B**

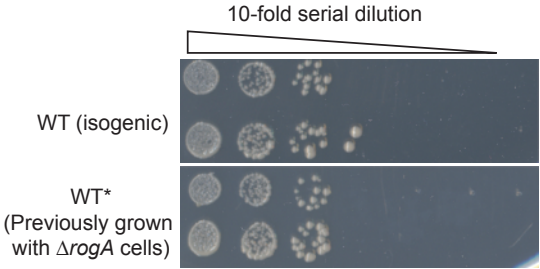

**C**

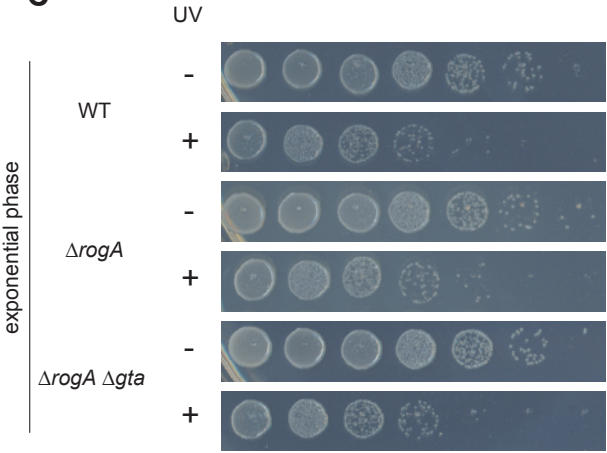

Supplement: S6 Fig — (A) Survival of the wild-type (hfaB::kanR) strain in combination with WT, ΔrogA, ΔrogA ΔdriD, or ΔdriD cells at 48 h and 96 h in stationary phase. (B) Survival after 96 h of representative replicates of either an isogenic WT strain or of surviving WT cells that had previously co-incubated with ΔrogA cells for 96 h. (C) Survival of exponential WT, ΔrogA, or ΔrogA Δgta cells to UV exposure (1 J/m2). (PDF) [file pbio.3001790.s006.pdf]

Fig. 2A

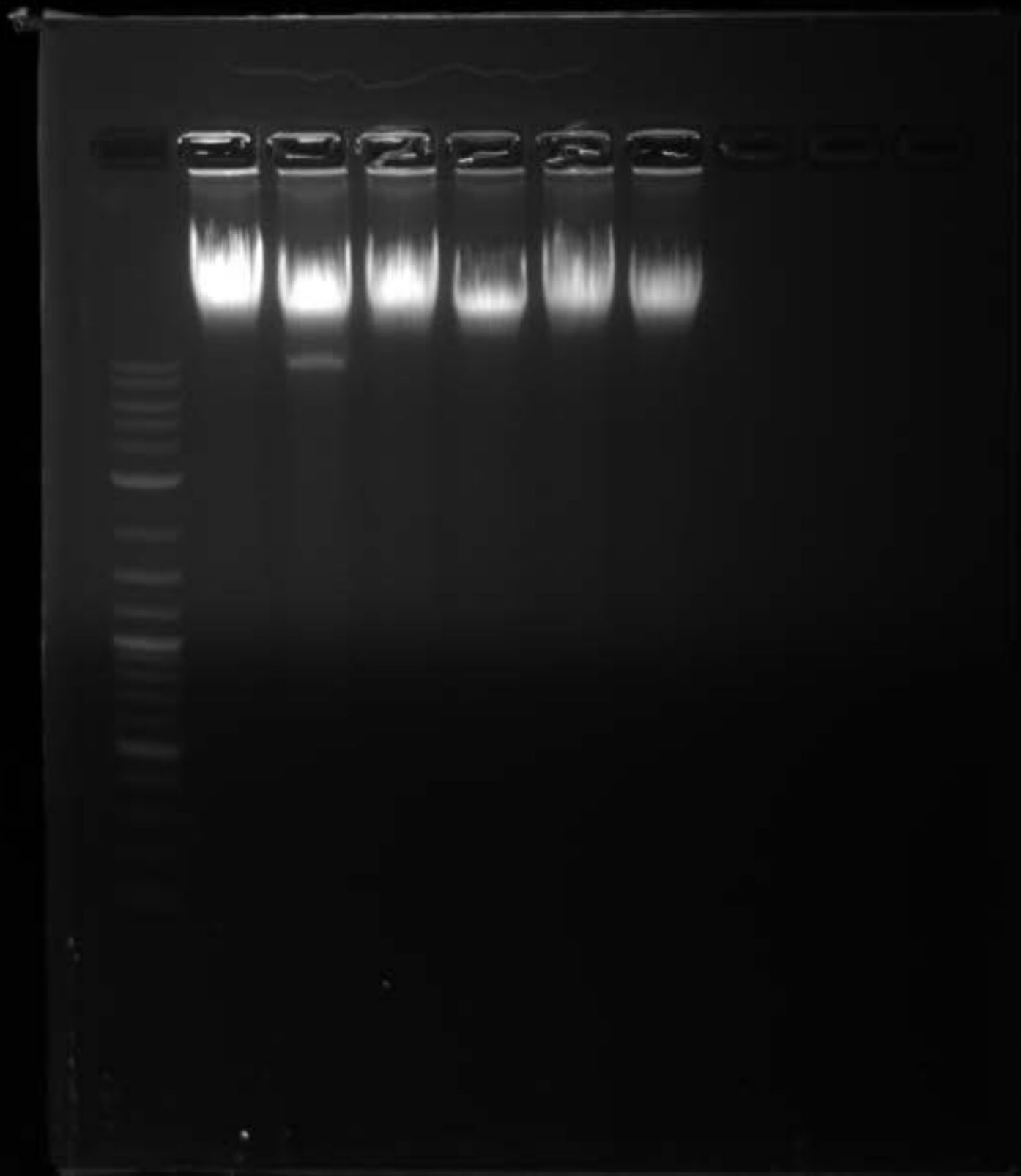

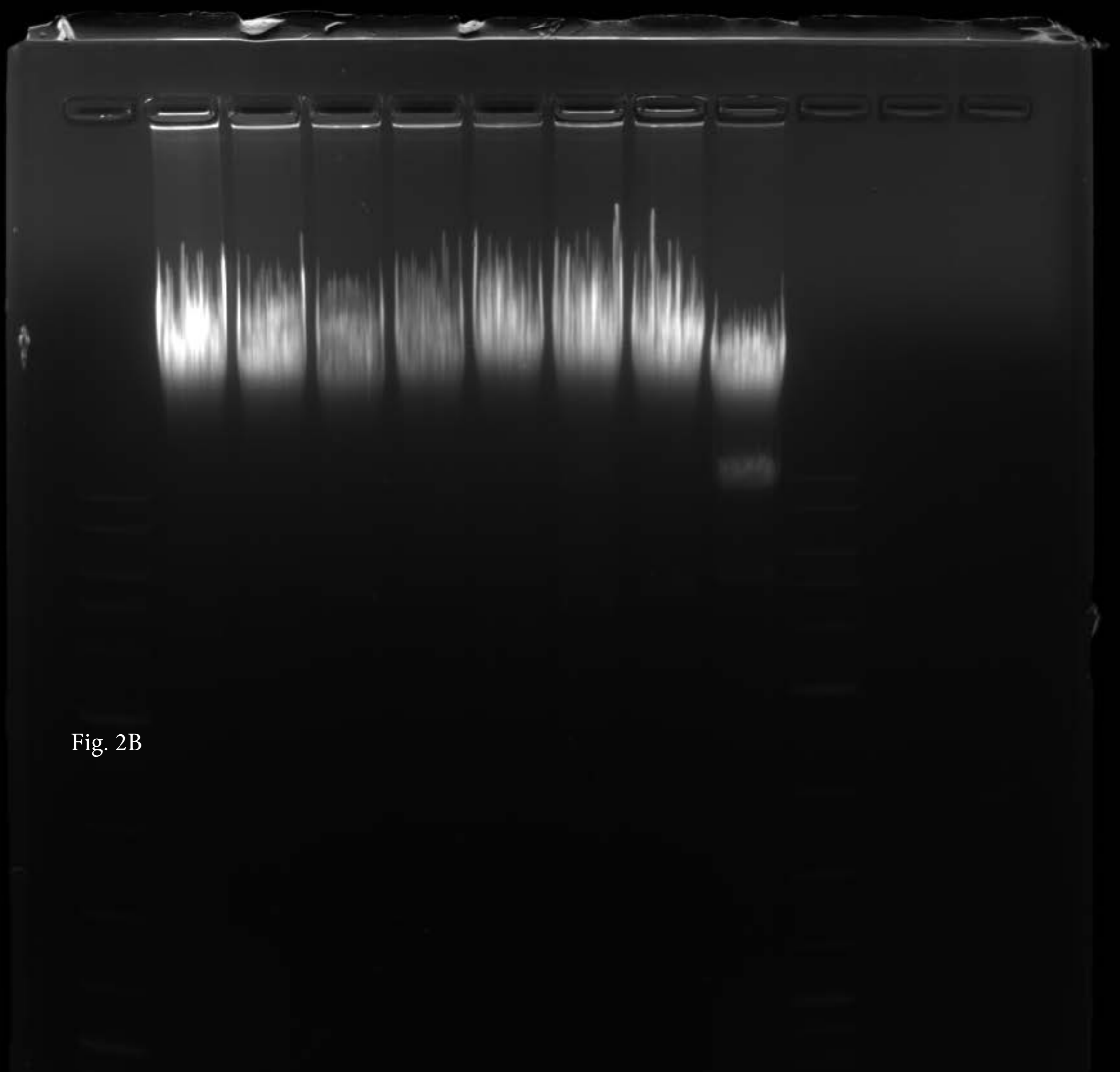

Fig. 2B

Fig. 2G FLAG input

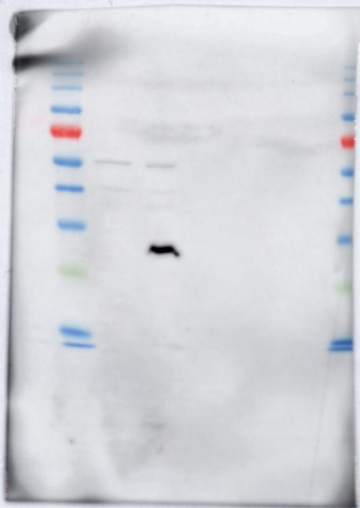

Fig. 2G - ParB input + IP

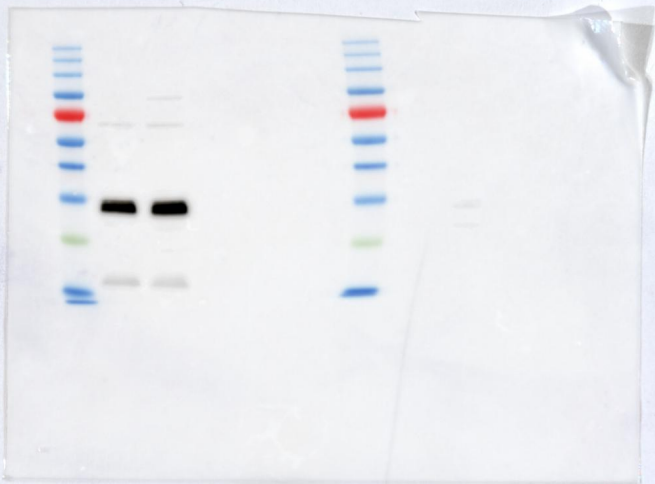

Fig. 2G- GafY input + IP

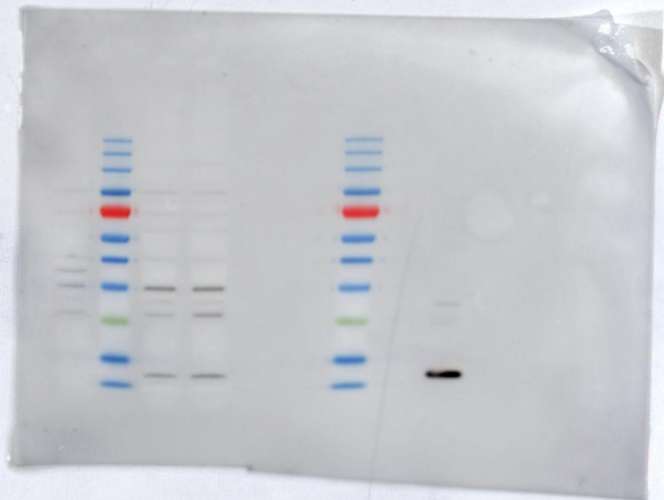

Fig. 2G - FLAG IP

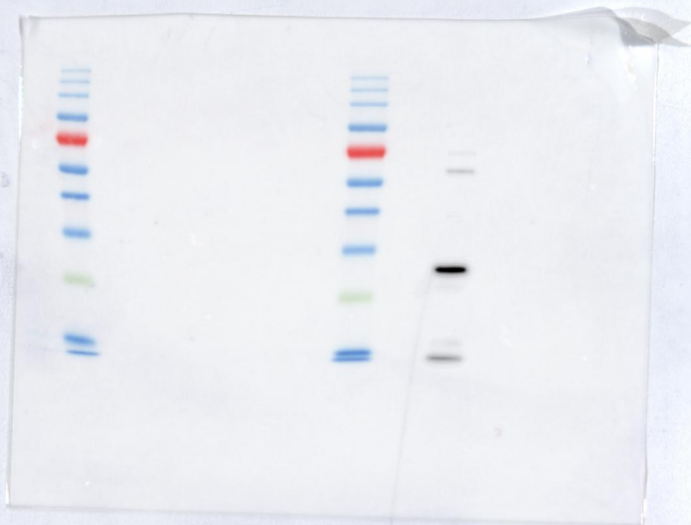

Supplement: S1 Raw Images — (PDF) [file pbio.3001790.s011.pdf]

Fig. S5F

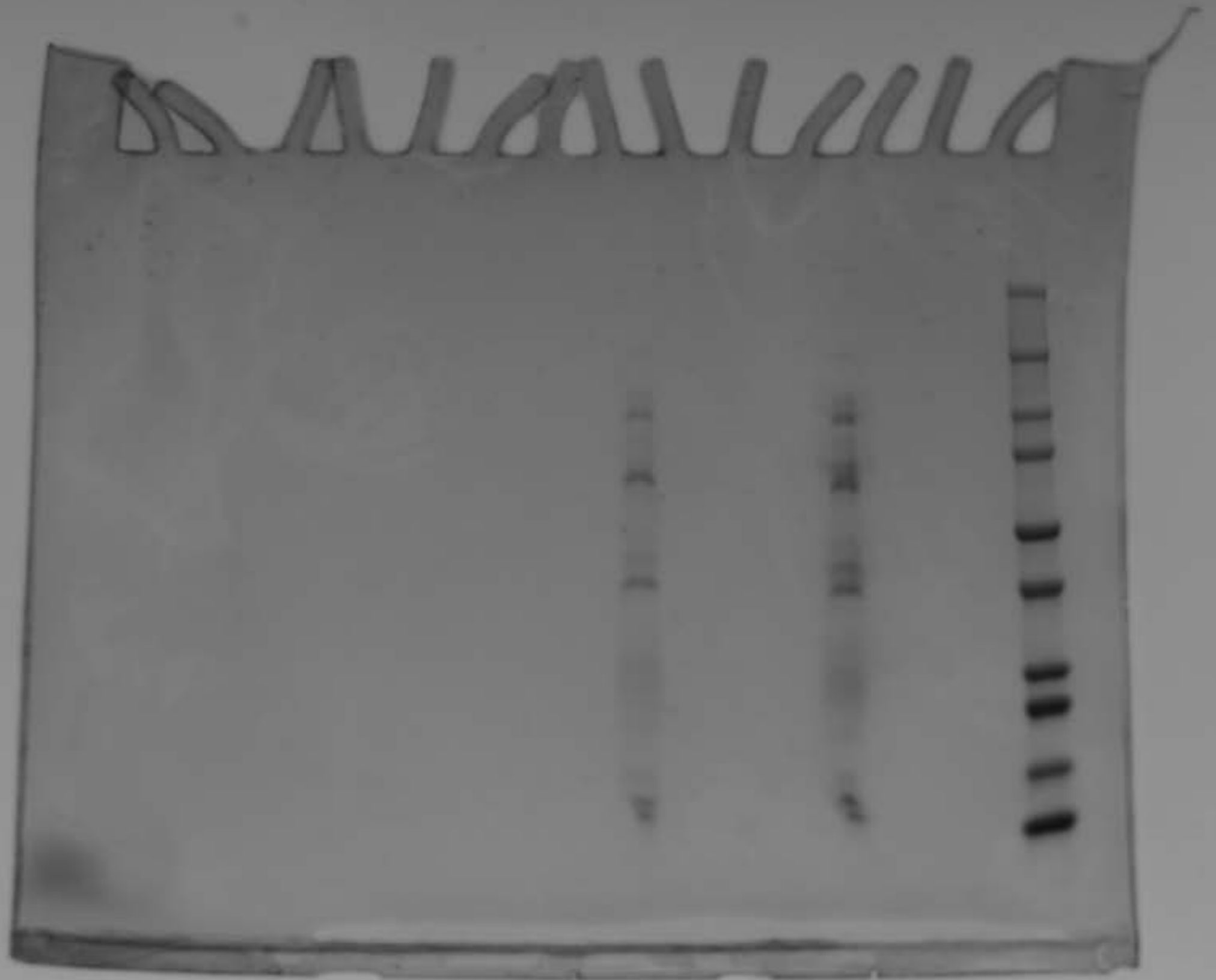

Fig. S5A

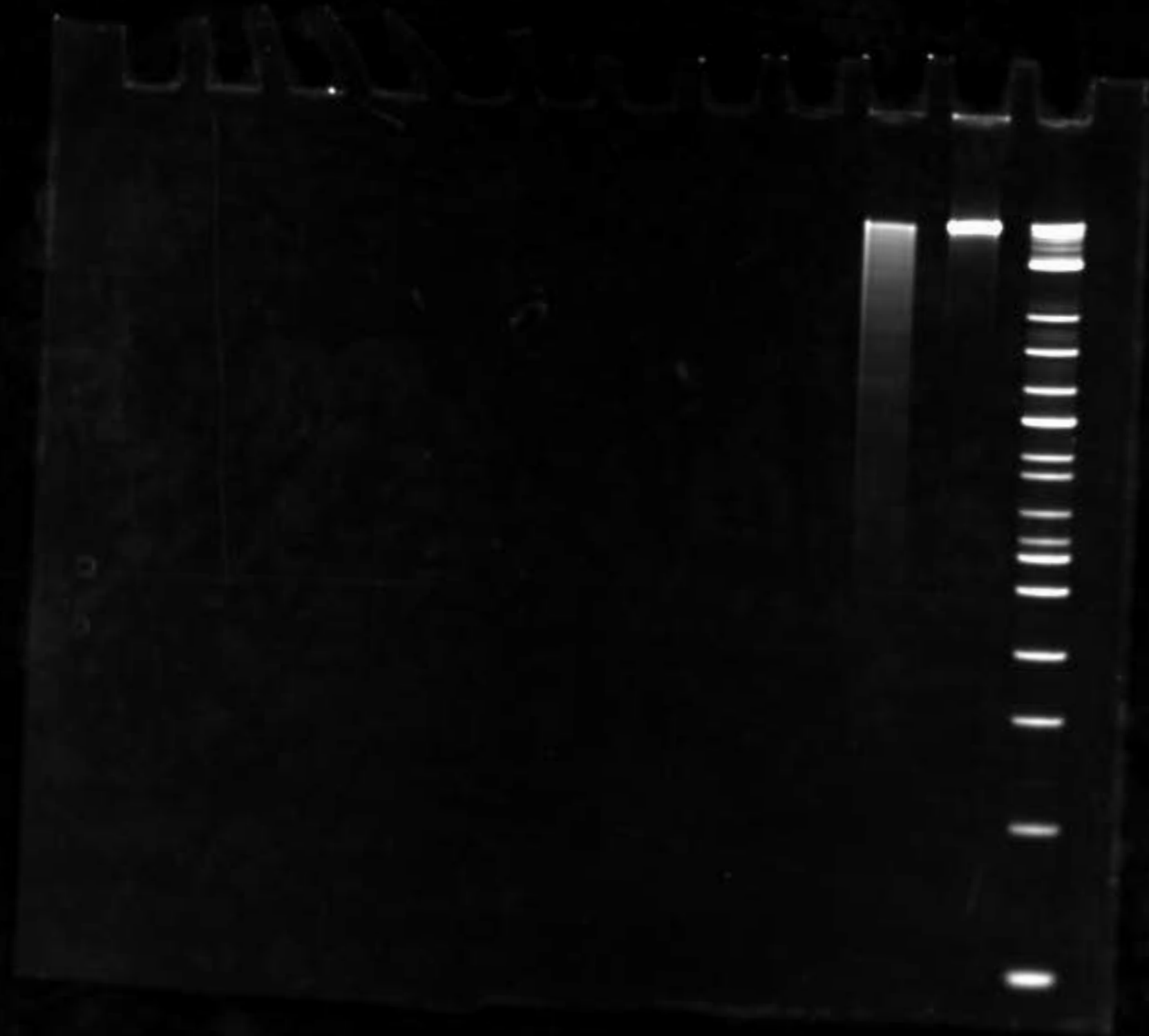

Supplement: S2 Raw Images — (PDF) [file pbio.3001790.s012.pdf]
