# Supplementary material for: Prophage-like gene transfer agents promote Caulobacter crescentus survival and DNA repair during stationary phase
Source: PLoS Biol. 2022 Nov 3;20(11):e3001790. doi: 10.1371/journal.pbio.3001790 (PMC9632790; doi:10.1371/journal.pbio.3001790)
Supplement: S2 Fig — (A) Comparison of the main GTA clusters from Caulobacter crescentus NA1000 and Rhodobacter capsulatus SB1003. The corresponding homolog of each C. crescentus gene in R. capsulatus is shown with the same color. (B) Amino acid sequence alignment of GafY and GafZ from C. crescentus to GafA in R. capsulatus. Identical amino acids are shaded black, with similar amino acids shaded in gray. (C) Co-occurrence of GTA cluster genes (all genes >100 AA), gafY, gafZ, rogA, and the GTA tail fiber homolog found across the α-proteobacteria. Co-occurrence is shown as the (# of genomes with at least 1 homolog of both gene X and gene Y)/(# of genomes with a homolog of gene Y). Non-symmetry across the diagonal plane results from the change in # of genomes in the denominator. Data are available in S1 Data. (D) β-galactosidase assay measuring transcriptional activity of a didA reporter (PdidA-lacZ and ΔdidA) bearing a high-copy plasmid with the xylose promoter driving expression of either nothing (empty), gafY alone, or both gafY and gafZ in their native arrangement. Cells were grown up to stationary phase (OD600 = ~1.3) and induced with or without xylose for 3 h. (n = 3, error bars indicate SD). * = p-value <0.05 of indicated comparisons. Data are available in S1 Data. (PDF) [file pbio.3001790.s002.pdf]

Figure S2

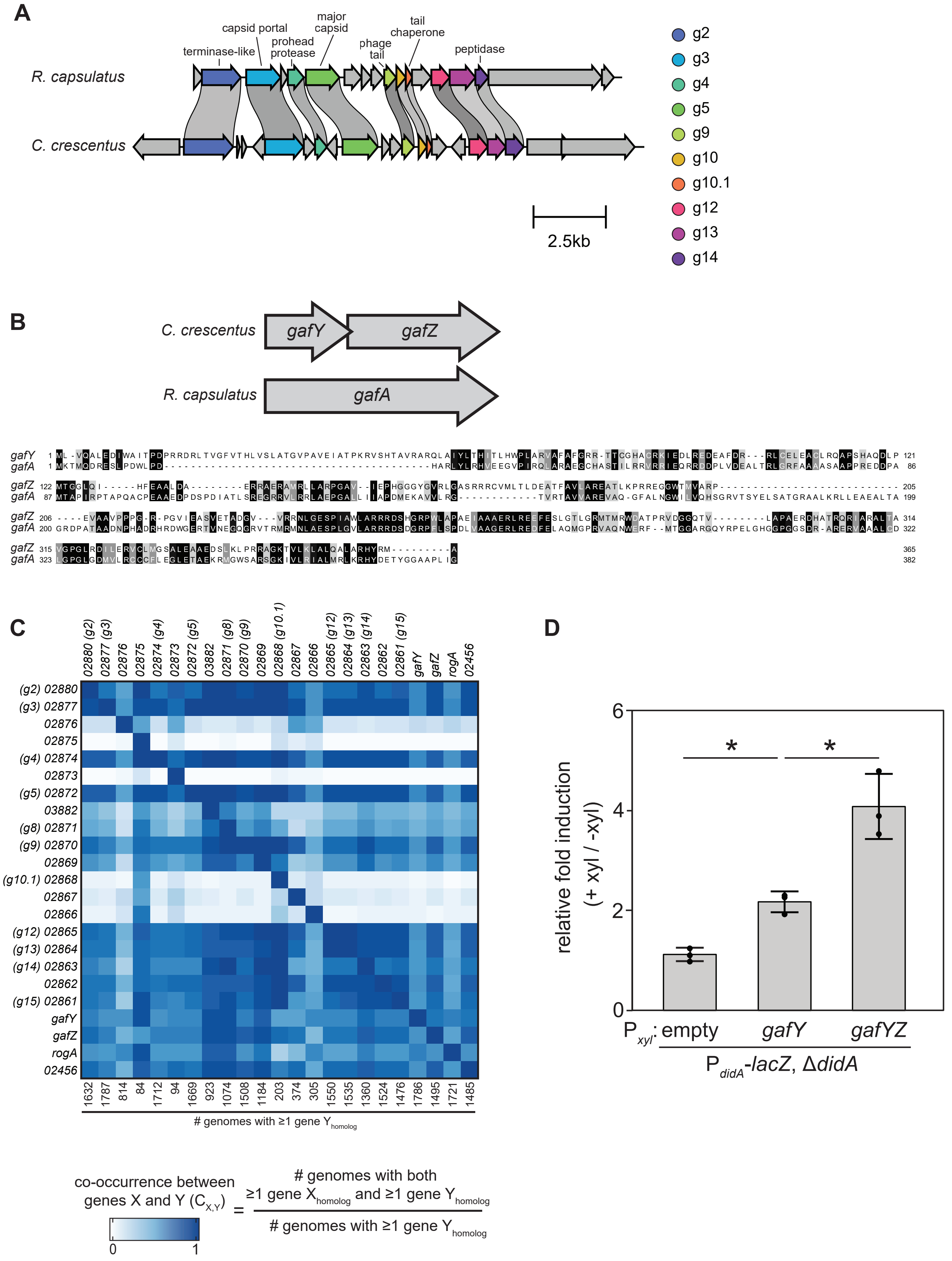
A
terminase-like
capsid portal
prohead protease
major capsid
phage tail
tail chaperone
peptidase
R. capsulatus
C. crescentus
g2
g3
g4
g5
g9
g10
g10.1
g12
g13
g14
2.5kb
B
C. crescentus
gafY
gafZ
R. capsulatus
gafA
C
02880 (g2)
02877 (g3)
02876
02875
02874 (g4)
02873
02872 (g5)
03882
02871 (g8)
02870 (g9)
02869
02868 (g10.1)
02867
02866
02865 (g12)
02864 (g13)
02863 (g14)
02862
02861 (g15)
gafY
gafZ
rogA
02456
1632 1787 814 84 1712 94 1669 923 1074 1508 1184 203 374 305 1550 1535 1360 1524 1476 1786 1495 1721 1485
genomes with ≥1 gene Y_homolog
co-occurrence between genes X and Y (C_X,Y) = # genomes with both ≥1 gene X_homolog and ≥1 gene Y_homolog / # genomes with ≥1 gene Y_homolog
0
1
D
relative fold induction (+ xyl / -xyl)
0
2
4
6
*
*
P_xyl: empty
gafY
gafYZ
P_didA-lacZ, ΔdidA
