# Supplementary material for: Prophage-like gene transfer agents promote Caulobacter crescentus survival and DNA repair during stationary phase
Source: PLoS Biol. 2022 Nov 3;20(11):e3001790. doi: 10.1371/journal.pbio.3001790 (PMC9632790; doi:10.1371/journal.pbio.3001790)
Supplement: S1 Table — (PDF) [file pbio.3001790.s007.pdf]

Table S1. Plasmids

Plasmid	Description	Source
pBXMCS-2::P_{xyl}-*gafY*	pBXMCS-2 transcribing *gafY*	This study
pBXMCS-2::P_{xyl}-*gafZ*	pBXMCS-2 transcribing *gafZ*	This study
pBXMCS-2::P_{xyl}-*gafYZ*	pBXMCS-2 transcribing *gafYZ*	This study
pRVMCS-2::P_{rogA}-*rogA*	low copy vector for expressing *rogA* from its native promoter for complementation	This study
pNPTS138::*tet*^{R} *1.0Mb*	used for the insertion of a tetracycline resistance marker near 1.0Mb position of chromosome	This study
pNPTS138::*tet*^{R} *2.0Mb*	used for the insertion of a tetracycline resistance marker near 2.0Mb position of chromosome	This study
pNPTS138::Δ*rogA*	used to create a markerless in-frame *rogA* deletion	This study
pNPTS138::Δ*rogA*::*tet*^{R}	used to create a tetracycline resistance-marked *rogA* deletion, maintaining the first 9 nucleotides of the *rogA* ORF	This study
pNPTS138::Δ*gta*	used to create a deletion of the GTA cluster from *CCNA_02880* to *CCNA_02861*	This study
pNPTS138::Δ*gta*::*kan*^{R}	used to create a kanamycin-resistance marked deletion of the GTA cluster from *CCNA_02880* to *CCNA_02861*	This study
pNPTS138::*hfaB*::*kan*^{R}	used to create a kanamycin-resistance marker at the *hfaB* locus on the chromosome	This study
pNPTS138::Δ*gafY*	used to create a markerless in-frame *gafY* deletion	This study
pNPTS138::Δ*gafZ*	used to create a markerless in-frame *gafZ* deletion	This study
pNPTS138::Δ*gafYZ*	used to create a markerless in-frame *gafYZ* deletion	This study
pNPT-spec-Δ*driD*	used to create a markerless in-frame *driD* deletion (*spec*^{R})	Modell *et al.* 2014
pNPTS138::*flag-gafZ*	Plasmid to insert a FLAG tag to the N-terminus of GAFZ	This study
pNPTS138::Δ*gafY*::*8xTAG*	Plasmid to insert 8 stop codons (TAG) 10 codons downstream of the start codon of *gafY*, thereby inactivating *gafY* without affecting the expression of the downstream *gafZ*	This study
pNPTS138::Δ*rogA*::*tet*^{R} *2*	used to create a tetracycline resistance-marked *rogA* deletion, maintaining the first 30 nucleotides of the *rogA* ORF	This study
pNPTS138::Δ*CCNA_02880*	used to create a markerless in-frame deletion of *CCNA_02880*	This study
pNPTS138::Δ*CCNA_02877*	used to create a markerless in-frame deletion of *CCNA_02877*	This study
pNPTS138::Δ*CCNA_02872*	used to create a markerless in-frame deletion of *CCNA_02872*	This study
pNPTS138::Δ*CCNA_02873*	used to create a markerless in-frame deletion of *CCNA_02873*	This study
pENTR::*gafY*	Entry plasmid that harbors the coding sequence of GafY, *kan*^{R}	This study
pML333	Gateway-cloning destination plasmid for fusing the protein interest to an N-terminally His_6-MBP tag, IPTG-inducible T7 promoter, *carb*^{R}	Laub lab collection
pML333::*gafY*	Plasmid for overexpression of a His_6-MBP-GafY	This study
pET21b::*rogA*	Plasmid for overexpression of RogA-His_6, *carb*^{R}	This study
pCOLADuet-1	Plasmid for co-overexpression of two proteins in *E. coli*, IPTG-inducible T7 promoter, *kan*^{R}	Merck
pCOLADuet-1:*his6-gafZ*	Plasmid for overexpression of His_6-GafZ in *E. coli*, *kan*^{R}	This study
pCOLADuet-1:*his6-gafZ gafY*	Plasmid for co-overexpression of His_6-GafZ and tagless GafY in *E. coli*, *kan*^{R}	This study
pBXMCS-2	medium-high copy plasmid containing a multiple cloning site downstream of the P_{xyl} promoter (*kan*^{R})	Thanbichler *et al.* 2007
pRVMCS-2	used as a low-copy vector backbone for plasmid-borne complementation (*kan*^{R})	Thanbichler *et al.* 2007
pXGFPC-2	used for the insertion of a kanamycin resistance marker on the chromosome (*kan*^{R})	Thanbichler *et al.* 2007
pXGFPC-6	used for the insertion of a chloramphenicol resistance marker on the chromosome (*chl*^{R})	Thanbichler *et al.* 2007
pNPTS138	integrating vector used to perform deletions and allelic replacements (*kan*^{R})	Dickon Alley lab collection
pENTR-D-TOPO	ENTRY vector for Gateway cloning, *kan*^{R}	Invitrogen

References

1. Modell JW, Kambara TK, Perchuk BS, Laub MT. A DNA damage-induced, SOS-independent checkpoint regulates cell division in *Caulobacter crescentus*. PLoS Biol. 2014;12(10):e1001977.

2. Thanbichler M, Iniesta AA, Shapiro L. A comprehensive set of plasmids for vanillate - and xylose-inducible gene expression in *Caulobacter crescentus*. Nucleic Acids Res. 2007;35(20):e137.
