# Supplementary material for: Prophage-like gene transfer agents promote Caulobacter crescentus survival and DNA repair during stationary phase
Source: PLoS Biol. 2022 Nov 3;20(11):e3001790. doi: 10.1371/journal.pbio.3001790 (PMC9632790; doi:10.1371/journal.pbio.3001790)
Supplement: S2 Table — (PDF) [file pbio.3001790.s008.pdf]

Table S2. Strains

Strain	Genotype	Source
ML76	CB15N	Evinger and Agabian (1977)
ML77	CB15N *rec526*	O'Neill *et al.* (1985)
ML2169	CB15N Δ*didA hfaB*-P$_{didA}$-*lacZ-hfaB*	Modell *et al.* (2014)
ML3660	CB15N Δ*rogA*::*tet*^{R}	This study
ML3661	CB15N Δ*rogA*::*tet*^{R} Δ*didA hfaB*-P$_{didA}$-*lacZ-hfaB*	This study
ML3662	CB15N Δ*rogA*::*tet*^{R} Δ*didA hfaB*-P$_{didA}$-*lacZ-hfaB* Δ*driD*	This study
ML3663	CB15N Δ*rogA*::*tet*^{R} Δ*didA hfaB*-P$_{didA}$-*lacZ-hfaB* + pRVMCS-2::P$_{rogA}$-*rogA*	This study
ML3664	CB15N Δ*rogA*::*tet*^{R} + pRVMCS-2::P$_{rogA}$-*rogA*	This study
ML3665	CB15N Δ*rogA*::*tet*^{R} Δ*driD*	This study
ML3666	CB15N Δ*rogA*::*tet*^{R} Δ*gta*::*kan*^{R}	This study
ML3667	CB15N Δ*rogA*::*tet*^{R} Δ*gafY*	This study
ML3668	CB15N Δ*rogA*::*tet*^{R} Δ*gafZ*	This study
ML3669	CB15N Δ*rogA*::*tet*^{R} Δ*gafYZ*	This study
ML3670	CB15N pBXMCS-2 (*kan*^{R})::P$_{xyl}$-empty	This study
ML3671	CB15N pBXMCS-2 (*kan*^{R})::P$_{xyl}$-*gafY*	This study
ML3672	CB15N pBXMCS-2 (*kan*^{R})::P$_{xyl}$-*gafZ*	This study
ML3673	CB15N pBXMCS-2 (*kan*^{R})::P$_{xyl}$-*gafYZ*	This study
ML3674	CB15N Δ*rogA*	This study
ML3675	CB15N Δ*gta*	This study
ML3676	CB15N Δ*gta* pBXMCS-2 (*kan*^{R})::P$_{xyl}$-*gafYZ*	This study
ML3677	CB15N *tet*^{R} *1.0Mb* pBXMCS-2 (*kan*^{R})::P$_{xyl}$-*gafYZ*	This study
ML3678	CB15N *tet*^{R} *2.0Mb* pBXMCS-2 (*kan*^{R})::P$_{xyl}$-*gafYZ*	This study
ML3679	CB15N pXGFPC-6 integrated *hfaB*::*kan*^{R}	This study
ML3680	CB15N I-SceI site and *tet*^{R} at *CCNA_00727* locus P$_{van}$-*i-SceI*, *chl*^{R} at *van* locus Δ*didA* *hfaB*::P$_{didA}$-*lacZ*	This study
ML3681	CB15N I-SceI site and *tet*^{R} at *CCNA_00727* locus P$_{van}$-*i-SceI*, *chl*^{R} at *van* locus Δ*didA* *hfaB*::P$_{didA}$-*lacZ* pBXMCS-2 (*kan*^{R})::P$_{xyl}$-empty	This study
ML3682	CB15N Δ*didA hfaB*-P$_{didA}$-*lacZ-hfaB* pBXMCS-2 (*kan*^{R})::P$_{xyl}$-empty	This study
ML3683	CB15N Δ*didA hfaB*-P$_{didA}$-*lacZ-hfaB* pBXMCS-2 (*kan*^{R})::P$_{xyl}$-*gafY*	This study
ML3685	CB15N Δ*didA hfaB*-P$_{didA}$-*lacZ-hfaB* pBXMCS-2 (*kan*^{R})::P$_{xyl}$-*gafYZ*	This study
ML3769	CB15N Δ*driD*::*tet*^{R}, Δ*didA hfaB*::P$_{didA}$-*lacZ*	Gozzi *et al.* (2022)
NTS2275	CB15N Δ*rogA*::*tet*^{R} 2	This study
NTS2481	CB15N *gafZ*::*flag-gafZ*	This study
NTS2501	CB15N Δ*gafY*::markerless	This study
NTS2515	CB15N Δ*rogA*::*tet*^{R} 2 Δ*gafY*	This study
NTS2489	CB15N Δ*rogA*::*tet*^{R} 2 *gafZ*::*flag-gafZ*	This study
NTS2316	CB15N Δ*rogA*::*tet*^{R} 2 Δ*CCNA_02880*	This study
NTS2445	CB15N Δ*rogA*::*tet*^{R} 2 Δ*CCNA_02877*	This study
NTS2314	CB15N Δ*rogA*::*tet*^{R} 2 Δ*CCNA_02872*	This study
NTS2403	CB15N Δ*rogA*::*tet*^{R} 2 Δ*CCNA_02873*	This study
DH5α	general *E. coli* cloning strain	Invitrogen
Rosetta (DE3)	*E. coli* host for protein overexpression from an IPTG-inducible T7 promoter F- *ompT hsdSB*(rB- mB-) *gal dcm* (DE3) pRARE (*chl*^{R})	Merck

References

1. Evinger M, Agabian N. J Bacteriol. 1977; 132(1):294-301.

2. O'Neill EA, Hynes RH, Bender RA. Mol Gen Genet. 1985; 198(2)275-8.

3. Modell JW, Kambara TK, Perchuk BS, Laub MT. PLoS Biol. 2014;12(10):e1001977.

4. Gozzi KG, Salinas R, Nguyen VD, Laub MT, Schumacher MA. Genes Dev. 2022; 36(9-10):618-633.
