# Supplementary material for: Prophage-like gene transfer agents promote Caulobacter crescentus survival and DNA repair during stationary phase
Source: PLoS Biol. 2022 Nov 3;20(11):e3001790. doi: 10.1371/journal.pbio.3001790 (PMC9632790; doi:10.1371/journal.pbio.3001790)
Supplement: S3 Table — (PDF) [file pbio.3001790.s009.pdf]

Table S3. DNA oligonucleotides

Oligo	Sequence	Source
oKRG92	gatccatATGCTGGTTCAAGCCCTTGAGG	This study
oKRG93	gatcGAGCTCgcccgccggtcaTTACGGCAG	This study
oKRG400	gatcCATatgaccggcgggcttcagatcc	This study
oKRG401	gatcGAGCTCcgactgaacgtcgcctcatgccat	This study
oKRG402	gaaatggatctgaagcccgccggtcaTgctCGGCAGATCCTGAGCGTGCGACG	This study
oKRG403	Atgaccggcgggcttcagatccatttc	This study
oKRG446	cagcagGAATTCcGCTTTTCCATCTGCCAGATCCAGTAG	This study
oKRG447	GGACCGAGAAAGGGTGACGATTGAGACCCTCGCGCCGTTTGGGCTTAG	This study
oKRG448	gcaacgagccgatcgctgatATTCCCTGCGTGCCTAACATGTTAG	This study
oKRG449	cagcagGTCGACgCACAGCGCGAACGGCGCGATC	This study
oKRG458	cagcagGAATTCcGAGGAGCTTATTGAAGCGCGCGAGC	This study
oKRG459	GGACCGAGAAAGGGTGACGATTGACCTTCCGGTGGCGGCCAAACC	This study
oKRG460	gcaacgagccgatcgctgatCGGAATGGCGCGCCGAGGCGATTG	This study
oKRG461	cagcagGTCGACgGGATGTCGAGAACGCCTTCGTCAGCC	This study
oKRG103	GGTCGCCATCGGCTCCTCC	This study
oKRG104	ggaggagccgatggcgaccAGCCAGTAGGCCGACCCGAC	This study
oKRG105	cagcagcttaagAAAACCCACGGCTTCGACCC	This study
oKRG102	cagcagactagtACTTGCCCAGCATCGCTTCG	This study
oKRG106	GGACCGAGAAAGGGTGACGATTGAGGTCGCCATCGGCTCCTCC	This study
oKRG107	gcaacgagccgatcgctgatAGCCAGTAGGCCGACCCGAC	This study
oKRG440	TCAATCGTCACCCTTTCTCGGTCC	This study
oKRG441	atcagcgatcggctcgttgc	This study
oKRG111	cagcaggaattcGAACGCTTCGGCCGCCAG	This study
oKRG110	gcgtttgcgcagatgcgcTTTGGGCTCAGGGACCATCG	This study
oKRG109	GCGCATCTGCGCAAACGC	This study
oKRG108	cagcaggaattcTCGATCTGGCCAAGGTCGAATTTC	This study
oKRG112	cagcagactagtTGTCCTGGTTCGACGTCACAGC	This study
oKRG113	ACACCGACGGCCTGAAGGG	This study
oKRG114	GGATGTTGTAGTTCAGCTCGGTGATG	This study
oKRG115	cagcagcttaagAACAATACCGTCATCGTCAATTCCAGC	This study
oKRG116	cccttcaggccgtcggtgtGCCAGTGTTACAACCAATTAACCAATTCTG	This study
oKRG117	CATCACCGAGCTGAACTACAACATCCAAAGCCACGTTGTGTCTCAAAATCTCTG	This study
oKRG118	cagcaggaattcGGGTGAAGGCCGTGGACACG	This study
oKRG119	AACCAGCATggcgtcctctcctcc	This study
oKRG120	ggaggagaggacgccATGCTGGTT CTGCCGTAAtgaccggcgggc	This study
oKRG121	cagcaggaattccgtggcgtcccatcgcatgg	This study
oKRG122	cagcaggaattcGACCTAGGCGATGGAAAAAC	This study
oKRG123	GCCGGTCATTACGGCAGATC	This study
oKRG124	gatctgccgtaatgaccggcATGGCATGAGGCGACGTTCAGTCG	This study
oKRG125	cagcaggaattccGACTTCCCCGCCTGGTTCCG	This study
oKRG126	ggaggagaggacgccATGGCATGAGGCGACGTTCAGTCG	This study
oKRG127	gatcggcgccCATTGCACAGCTGGCGGATCG	This study
oKRG128	gatccttaagGGCCTACTGGCTGACCCAGACGATC	This study
oKRG523	CGTGCTGCGCAATCACAAGG	This study

oKRG524	GTTCGATACAAATGCGTAGACACGCC	This study
NTP2598	gcaattgaagccggctggcgccaATCCACAGAACCTCATGAGCGTAG	This study
NTP2599	CTTGTCGTCATCGTCTTTGTAGTCCATTACGGCAGATCCTGAGCGTGCG	This study
NTP2600	GACTACAAAGACGATGACGACAAGACCGGCGGGCTTCAGATCCATTTC	This study
NTP2601	cggccgaagctagcgaattcgtgACCGTTTGGCCCCCGTCGACGCG	This study
NTP2642	gcaattgaagccggctggcgccaTCACACACCGGAGGGCCGCACGG	This study
NTP2643	ctactactactactactactactaCCAGATGTCCTCAAGGGCTTGAAC	This study
NTP2644	tagtagtagtagtagtagtagtagGCGATCACGCCCGATCCCCGAC	This study
NTP2645	cggccgaagctagcgaattcgtgGAAGGTGGCCTCGTCCAGGGTCAG	This study
NTP2194	gcaattgaagccggctggcgccaGATGTCGTCGCGCTGTTGCTCC	This study
NTP2305	ggaccgagaaagggtgacgattgaCCAGATTTCGCCGTGAGACAG	This study
NTP2303	tcaatcgtcaccctttctcgg	This study
NTP2304	tcagcgatcggctcgttgccc	This study
NTP2306	cgcagggcaacgagccgatcgctgaAGCCAGTAGGCCGACCCGACAG	This study
NTP2197	cggccgaagctagcgaattcgtgGCGCTGATCGAGGAAAAGCTG	This study
NTP2230	gcaattgaagccggctggcgcca ACGCCGGCGAACAGGCGCAGC	This study
NTP2231	GCCCGCCAATCCCCATAGTC	This study
NTP2232	CTCGACTATGGGGATTGGCGGGC tag GTGGCCGAGGCCAACCAGGGC	This study
NTP2233	cggccgaagctagcgaattcgtgCACCTCTCCTCCCCCATCGGG	This study
NTP2493	gcaattgaagccggctggcgccaTGAGATCGCCGGAGGGGCTATG	This study
NTP2494	AGACGCCATGCAAACGCCAAGAG	This study
NTP2495	CTCTTGGCGTTTGCATGGCGTCTGAGCAGTAAAATCCTCCCCCCG	This study
NTP2496	cggccgaagctagcgaattcgtgGGCTCTGCGCAAAAGCGCCGG	This study
NTP2222	gcaattgaagccggctggcgccaTGATGATCGGGCCCAGGTCGC	This study
NTP2223	CTCTTTCATGGCGTTCTCCGAAG	This study
NTP2224	CTTCGGAGAACGCCATGAAAGAG GCGTCGTAATCCAATCCTCCC	This study
NTP2225	cggccgaagctagcgaattcgtgCGCTGACCGGCGATGGCGGTG	This study
NTP2323	gcaattgaagccggctggcgccaGACAGACCCTTGGCTTCCAGC	This study
NTP2324	GTCGGTCATTTCGTCAGCTTCG	This study
NTP2325	GCGAAGCTGACGAAATGACCGACGACCCGGACGGGACGCTGCTAAG	This study
NTP2326	cggccgaagctagcgaattcgtgGGATCGACGAGATGCAGCGC	This study
NTP2568	ggctccgcggccgccccccttcacc ATGCTGGTTCAAGCCCTTGAG	This study
NTP2569	agctgggtcggcgcgcccacccttttaTTACGGCAGATCCTGAGCGTG	This study
NTP2291	tttaactttaagaaggagatatacatatgGCGACCCTGTCTCACGGCG	This study
NTP2292	gtggtgctcgagtgcggccgcaagcttCTGGCTGACCCAGACGATCC	This study
NTP2693	ccatcatcaccacagccaggatccgAATTCGatgACCGGCGGGCTTCAGATCC	This study
NTP2694	tcgacttaagcattatgcggccgcaagcttTCATGCCATCCGGTAGTGTCGG	This study
NTP2691	agttaagtataagaaggagatatacatATGCTGGTTCAAGCCCTTGAGGAC	This study
NTP2692	agcggtttctttaccagactcgagggtaccTTACGGCAGATCCTGAGCGTGCG	This study
*02880*_1_F	cagaagctcgcccgtcagatgcgccatggtcgtcctgctggttatggtgtg	This study
*02880*_1_R	cacaccataaccagcaggacgaccatggcgcatctgacgggcgagcttctgcctaccctacgtcctcctgc	This study
*02880*_2_F	ctgctggttatggtgtggagccgctatatgggagcgctaacatgacatgtc	This study
*02880*_2_R	gacatgtcatgttagcgctcccatatagcggctccacaccataaccagcagcctaccctacgtcctcctgc	This study
*02880*_3_F	cgctaacatgacatgtccatgcgcgctgagaccaggatagccaatcgggcg	This study
*02880*_3_R	cgcccgattggctatcctggtctcagcgcgcatggacatgtcatgttagcgcctaccctacgtcctcctgc	This study
*02880*_4_F	ggatagccaatcgggcgcccccggcgggccggtttttgcggcgcgcttgcc	This study

*02880*_4_R	ggcaagcgcgccgcaaaaaccggcccgccgggggcgcccgattggctatcccctaccctacgtcctcctgc	This study
*02880*_5_F	tttgcggcgcgcttgcccgtcaggggcctcgactatggggattggcgggcc	This study
*02880*_5_R	ggcccgccaatccccatagtcgaggcccctgacgggcaagcgcgccgcaaacctaccctacgtcctcctgc	This study
*02880*_6_F	atggggattggcgggcctgaacgctctgaataagccaggcgaagtctcgcg	This study
*02880*_6_R	cgcgagacttcgcctggcttattcagagcgttcaggcccgccaatccccatcctaccctacgtcctcctgc	This study
*02880*_7_F	ccaggcgaagtctcgcgaaatcagcggcttgtcacttttacgcgcccgcc	This study
*02880*_7_R	ggcgggcgcgtaaaagtgacaagccgctgatttcgcgagacttcgcctggcctaccctacgtcctcctgc	This study
*02880*_8_F	acttttacgcgcccgccaatcatccgcgcccgctggcgccgtatctttgtg	This study
*02880*_8_R	cacaaagatacggcgccagcgggcgcggatgattggcgggcgcgtaaaagtcctaccctacgtcctcctgc	This study
*02880*_9_F	aatcatccgcgcccgctggcgccgtatctttgtgtcatggccggaagaagc	This study
*02880*_9_R	gcttcttccggccatgacacaaagatacggcgccagcgggcgcggatgattcctaccctacgtcctcctgc	This study
*gafY*_1_F	cgtcgggcggcggggcgctccgagggggcgatatcctgcgtt	This study
*gafY*_1_R	aacgcaggatatcgccccctcggagcgccccgccgcccgacgCCTACCCTACGTCCTCCTGC	This study
*gafY*_2_F	cgagggggcgatatcctgcgttctatccacagaacctcatga	This study
*gafY*_2_R	tcatgaggttctgtggatagaacgcaggatatcgccccctcgCCTACCCTACGTCCTCCTGC	This study
*gafY*_3_F	ttctatccacagaacctcatgagcgtagcggcgacctaggcg	This study
*gafY*_3_R	cgcctaggtcgccgctacgctcatgaggttctgtggatagaaCCTACCCTACGTCCTCCTGC	This study
*gafY*_4_F	gagcgtagcggcgacctaggcgatggaaaaacaagcgcttag	This study
*gafY*_4_R	ctaagcgcttgtttttccatcgcctaggtcgccgctacgctcCCTACCCTACGTCCTCCTGC	This study
*gafY*_5_F	cgatggaaaaacaagcgcttagccccgtgagacatgagcgga	This study
*gafY*_5_R	tccgctcatgtctcacggggctaagcgcttgtttttccatcgCCTACCCTACGTCCTCCTGC	This study
*gafY*_6_F	agccccgtgagacatgagcggaataggaatatcttcctcagt	This study
*gafY*_6_R	actgaggaagatattcctattccgctcatgtctcacggggctCCTACCCTACGTCCTCCTGC	This study
*gafY*_7_F	gaataggaatatcttcctcagtgcggaatataaacctatagt	This study
*gafY*_7_R	actataggtttatattccgcactgaggaagatattcctattcCCTACCCTACGTCCTCCTGC	This study
*gafY*_8_F	gtgcggaatataaacctatagtggtgttcgccggccgcgctc	This study
*gafY*_8_R	gagcgcggccggcgaacaccactataggtttatattccgcacCCTACCCTACGTCCTCCTGC	This study
*gafY*_9_F	gtggtgttcgccggccgcgctcgcggcctggaggagaggacg	This study
*gafY*_9_R	cgtcctctcctccaggccgcgagcgcggccggcgaacaccacCCTACCCTACGTCCTCCTGC	This study
*gafY*_10_F	tcgcggcctggaggagaggacgccatgctggttcaagcccttt	This study
*gafY*_10_R	aagggcttgaaccagcatggcgtcctctcctccaggccgcgaCCTACCCTACGTCCTCCTGC	This study
*gafY*_11_F	cgccatgctggttcaagcccttgaggacatctgggcgatcac	This study
*gafY*_11_R	gtgatcgcccagatgtcctcaagggcttgaaccagcatggcgCCTACCCTACGTCCTCCTGC	This study
