# Supplementary material for: Prophage-like gene transfer agents promote Caulobacter crescentus survival and DNA repair during stationary phase
Source: PLoS Biol. 2022 Nov 3;20(11):e3001790. doi: 10.1371/journal.pbio.3001790 (PMC9632790; doi:10.1371/journal.pbio.3001790)
Supplement: Attachment — Submitted filename: GTA responses to reviewers D04.pdf [file pbio.3001790.s013.pdf]

We thank the reviewers for their comments and questions about our manuscript. Below we respond to each question or comment and indicate, when appropriate, how the text and/or figures have been updated in response. Reviewer text is in black, our responses are in blue.

Reviewer #1: This manuscript describes a novel gene transfer agent (GTA) in Caulobacter, its characterization, and the test of its role. GTAs are intriguing elements, probably derived from phages, that package the DNA of dying cells in viral-like particles. These particles can inject the DNA in other cells. The role of these elements is yet unclear and only a handful of model systems are known. Here, a novel one is introduced. This novel system has many similarities with known systems: its locus is smaller than prophages, it is regulated by systems homologous to known ones, it packages small DNA fragments (~8 kb) more or less randomly, triggers cell lysis, and can transfer the DNA to cells of the same species. The first 5 pages of results describe these results and are an interesting and solid contribution to the specific domain of GTA research. The final results concerns the test of hypothesis for the role of GTAs and are of broader interest. My review focus on the latter. The authors consider 3 hypothesis. (1) GTAs allow cells to acquire and spread new beneficial alleles, (2) their production releases beneficial nutrients (3) GTAs allow cells to acquire and use wild-type DNA to repair or revert deleterious mutations.

1) The test of the first hypothesis is not convincing. By co-incubating strains and their DrogA mutants the authors test if this allows the former to extend survival in the stationary phase. The details presented in results are so succinct that I'm uncertain of where this is described in methods. There seems to be no evidence that in this setup there are adaptive mutations to be transferred. It is also unclear why testing only in stationary phase (elements are produced in these conditions but can disperse and infect in other conditions). It's not clear to me if one can conclude anything from the experiments. If this hypothesis is to be tested, there should be clear knowledge of an advantageous mutation to be transferred between cells. And there should be tests that this can or cannot take place under a range of experimental conditions.

We apologize for the succinct presentation. We have now included a more detailed description of the experiment performed in the Methods section (lines 817-830) and expanded the Results section (lines 309-319) to better articulate the logic of the experiment performed and how to interpret it, which we summarize here as well. We believe the experiment is, in fact, convincing and clear cut. When WT cells are co-incubated with Δ*rogA* cells (GTA producers), there is a large, 10-30 fold increase in survival of the WT cells that is GTA-dependent (see Fig. 5B). It is well known that bacteria can acquire mutations that improve their survival in stationary phase (*e.g.* GASP mutants). So when we observed a 10-30 fold increase in survival of the WT cells, one reasonable hypothesis is that the GTA producers are enabling the distribution of an advantageous mutation. Critically, this hypothesis makes the strong prediction that if the GTAs were, in fact, providing an advantageous mutation, it would be a genetically heritable advantage. Thus, it is logical to ask, as we did, whether the WT cells exposed to GTA producers could, when recultured on their own, grow better in stationary phase - but they did not (Fig. S6B). It is therefore a safe and logical conclusion that the benefit seen for WT cells co-incubated with GTA producers is NOT via the acquisition of a beneficial allele/mutation. Importantly, it is not

required to know in advance that there is a beneficial mutation in the GTA producers - the experiment is testing whether the presence of an advantageous mutation, if it were present in the GTA producers, can explain the >10-fold increase in survival of WT cells that we documented. But the results show this is not the case, allowing us to rule out this first model/hypothesis.

We would also note that the immediately preceding sub-section in the Results (also see Fig. 4C) had already demonstrated that if a beneficial mutation, in that case a *tet*^{R} cassettee, were present in GTA producers it can be transferred and detected in recipient cells following selection. Thus, taken together, our results clearly rule out the notion that GTAs provide a benefit by transferring advantageous mutations.

With regard to testing this first hypothesis under a range of experimental conditions: we only observed the benefit arise in deep stationary phase (see Fig. 5C). It was not possible to accurately test the co-incubation of wild-type cells with GTA producers in exponential phase because GTA production only occurs in stationary phase, and adding stationary phase culture or supernatant to exponentially growing wild-type cells would induce entry to stationary phase, confounding the experiments. We did test the impact of GTAs during exponential versus stationary phase when examining survival following DNA damage and saw a benefit during stationary phase following UV (Fig. 5E and S6C, discussed line 333-339) and MMC (Fig. 6C-D, discussed line 389-393) treatment.

2) It's unclear why GTA would be a strategy to release nutrients. Viral particles do not enter the cell and they are very costly for the producer. Just the delivery of DNA does not seem an interesting strategy to feed other bacteria. The hypothesis seems not to work, so this is a minor detail.

We fully agree that it's not clear *a priori* why GTAs would be a useful strategy to release nutrients. In fact, we deemed this an extremely unlikely explanation *a priori*. But our goal was to try and enumerate and then test *all possible* explanations for the finding that WT cells co-incubated with GTA producers survive 10-30 fold better than cells incubated without the GTA producers. The key experiment performed to rule out the 'nutrient' hypothesis is presented on p. 12 of the manuscript. As stated there, if GTAs were either directly providing DNA as a food source (or if the lysed producer cells were providing nutrients), the benefit of co-culturing WT cells with GTA producers should be recombination independent. However, quite strikingly, *rec-* cells did not benefit from co-incubation with GTA producers the way WT cells did indicating that recombination *is* required for the benefit. Again, our goal was to try and systematically enumerate and test the *possible* models for the benefit seen for WT cells regardless of how likely they may seem *a priori*.

3)The authors conclude that their results "suggest that GTAs can improve a recipient cell's ability to survive DNA damage ". I agree with the conclusion that they can. However, I was surprised that they failed to mention similar tests of the broader hypothesis that uptake of conspecific DNA favors DNA repair. This is one of the most studied hypotheses for the existence of natural transformation and has been reviewed several times before (for example Claverys, Annual Rev Microbiol, 06). There are also several studies showing that DNA damaging agents promote

transformation hinting at a connection between acquisition of DNA and DNA repair. The results presented here should be integrated in the context of this literature. It should also be added that the hypothesis DNA for repair has been criticized on several other grounds, notably the likelihood that the incoming DNA matches the region in need of repair, which seems low since the genome is several Mb and the GTA carries less than 10 kb. Maybe the present results could add interesting facts to the existing debate, but at this stage that's unclear.

We thank the reviewer for pointing out the potential similarities of our findings with GTAs and those generated previously with natural transformation. We were certainly aware of this literature, but felt that it was somewhat dangerous/speculative to implicitly or explicitly equate GTAs and natural transformation, which differ in substantial ways. Perhaps most notable is the fact that cells acquiring DNA by transformation cannot 'know' the origin of the incoming DNA and whether it is conspecific or not. In contrast, because GTAs are related to phages, they require a specific receptor on a recipient cell, which in principle enables a specificity to DNA acquisition that is not possible in transformation. Nevertheless, prompted by the reviewer's comments, we have now added discussion and comparison of GTAs and transformation, particularly in response to DNA damage (see the section beginning on line 437). As the reviewer notes, the literature has hinted at connections between DNA damage and DNA uptake. We think our work now adds an important demonstration of how one mechanism of DNA uptake, via GTAs, can enable recipient cells to survive DNA damage. This has not been demonstrated previously in studies of GTAs; even for transformation, the evidence that DNA uptake provides a large benefit (comparable to the 10-30 fold increase in survival we find for recipients of GTAs) is quite limited as near as we can tell, so we do think our paper adds in important ways to this broader field. Indeed, as Reviewer 2 notes, our finding that GTAs can enable recombination-based repair "*is particularly noteworthy because HR has long been speculated to be a potential benefit to cells from producing GTAs but has never been proven. The work has great scope and importance...*". We thank Reviewer 1 for pushing us to broaden the discussion of our work.

Finally, with regard to the probability that incoming DNA would match the region in need of repair: the reviewer raises a very good point here, but we think it is important to keep in mind that even if rare, a cell that manages to repair its lesion via an incoming GTA particle can then proliferate, just as spontaneous point mutations that enable a cell to survive a selection such as rif-treatment are also rare, but can nevertheless arise and then proliferate in a culture. The question becomes at what frequency do cells harboring a given lesion acquire DNA that they can use to repair that lesion. Answering this question will require a better understanding of (i) how many GTA particles are released by each producer cell and (ii) how many GTA particles can be taken up by recipient cells. Assessing both is well beyond the scope of this paper. But we would point to the result in Fig. 6A that empirically demonstrates that repair *is* happening - our sequencing demonstrates that 12 of 14 recipient cells surviving after 72 hrs have replaced the marker gene next to the engineered I-SceI site indicating that they MUST have acquired WT DNA from GTAs. We think this is a remarkable and compelling result that will be of extremely broad interest.

-There is a theoretical complication specific to the GTAs. In transformation, bacteria program a response to acquire DNA eventually useful for repair. But in GTAs, the choice is not on the recipient, but on a bacterium that dies and offers its DNA. From the point of view of evolutionary biology this is much harder to explain for hypothesis #3 than for hypothesis #1. These issues are briefly mentioned in the discussion, but there is no integration with past works, no reference to models or literature, and this complicates the identification of the novelties in these results (beyond the fact that it deals with GTAs and not with other mechanisms of transfer).

We thank the reviewer for raising this important evolutionary angle to our work and we have now expanded on this topic in the Discussion (see lines 437-472).

-L69. "Modelling shows that it is more favourable for a population if cells that acquire a beneficial allele simply grow and propagate the allele via vertical inheritance rather than distribute the allele via GTAs. " This is given, if I understand correctly, as evidence that GTAs may not be adaptive for the transfer of novel functions. I find the argument very weak (if the cell is dying, saving some genes can be adaptive enough), but more importantly I don't see how the argument works better for the hypothesis #3 than for hypothesis #1. Why should bacteria carry elements whose death will allow the repair of DNA of potentially non-producer cells? If their genes are being reproduced (hypothesis #1) this seems easier to understand than if they are not (hypothesis #3).

The discussion of the possible ways of GTAs benefiting a population was not meant to be fully exhaustive. We had provided a brief discussion earlier on, where the reviewer mentioned, but we have now expanded the Discussion to include a richer evaluation of the possible benefits of GTAs (lines 437-472).

There is a pervasive lack of statistical analysis of the experimental results. Conclusions about differences (significant or non-significant) should be supported by statistical analyses.

We have now added statistical tests to bolster the conclusions we draw for the experiments in Figures 1C, 2E, 4E, 4F, 5A, 5D, 5E, 6D and S2C. We have also included a line regarding the statistical tests used throughout the manuscript in the Methods section (line 533-534).

-Figure 1 In C to test differences and in E for the threshold of fold change 4 (what does it meand in statistical terms).

Statistical analyses have been included for Fig. 1C. For Fig. 1E, all of the genes with a $\log_2$ fold change > 4 (16-fold) are also > 7 standard deviations away from the mean of the distribution, which we now note in the figure legend. Additionally, the induction of all these genes is comparable in the Δ*rogA* and *gafYZ* overexpression strains (Fig. 2E), with an R^2 value of 0.92 providing independent corroboration of their coordinate induction.

-Figure 2E has no statistics and one is informed that "Note only a portion of the plot is shown". That's not acceptable. The full plot should be in supplementary material and statistics are needed.

We now provide in Fig. 2E the full plot (with the R^2 value of 0.92 indicated in the figure legend) and have maintained the color-coding of the GTA cluster genes and regulators. Please also note

that all of the RNA-seq data were deposited in GEO and are fully available for download and analysis.

-Figure 4E needs stats.

We now include error bars and statistical analyses for Fig. 4F (formerly Fig. 4E).

-Same for figure 5 and 6.

These figures had error bars, which indicated statistically significant differences for the comparisons being made in the text, with large effect sizes as well. We now also provide notations in the figure indicating the high statistical significance of all comparisons made in the text.

-Figure S1C needs statistical analyses. At the very least one would need to know the denominator of the equation (the meaning of dark blue is very different if the latter is large or small). Statistical tests should also be corrected for phylogeny.

We have now included the denominator of the equation (# of genomes with ≥ 1 gene Y homolog) for each column on the bottom of the plot to provide more context for the cooccurrence calculation. We do not make any statements about statistical significance and are including this data as a simple reference for gene count co-occurrence across sequenced α-proteobacterial genomes.

Minor comments.

-L101. Unclear to me. If the regulator is LexA-like and its associated with response to DNA damage, why is it stated that it is SOS-independent? Maybe this is just a variant of the standard SOS response?

We do not state that RogA is SOS-independent. As noted at the beginning of this sub-section (lines 94-96), our prior work (ref 18; Modell et al 2014) demonstrated that DriD (not RogA) responds to DNA damage in a *recA*-independent manner indicating it is not part of the classic SOS response. Line 101 is referring to RogA, which is the new factor identified in this study. It does have some similarity to LexA, as noted in the text, but is not DNA damage regulated (either SOS-dependent or SOS-independent). It also lacks key residues required for cleavage of LexA and cI, which we now note in the revised text (line 106).

Reviewer #2 (Andrew S. Lang): The manuscript by Gozzi and colleagues presents a broad range of molecular and microbiology experiments that demonstrate the bacterium C. crescentus produces a gene transfer agent (GTA). They also demonstrate that production of this GTA leads to increased cell survival in the stationary phase of lab culture growth and that the GTAs allow DNA repair via homologous recombination. This is particularly noteworthy because HR has long been speculated to be a potential benefit to cells from producing GTAs but has never been proven. The work has great scope and importance, and the experiments are well designed and sound. The manuscript is well written, clear and has a good, logical flow.

One key (and simple) experiment that is missing, which is a definitive experiment to confirm that transformation is not somehow involved here, is the demonstration that the gene transfer still happens after the culture supernatant or filtrate is treated with DNase to degrade any free DNA released into the environment.

Thank you for the suggestion. We have now performed a GTA transfer assay with and without DNase and found no impact on transfer rates, suggesting that the transfer is DNase-independent (see new Fig. 4E, lines 265-269).

Is there any indication that there might be translational coupling for gafY and Z? I.e., could the gafY knockout be polar on gafZ?

To address this point, we tested whether a double *rogA gafY* deletion could be complemented by a low-copy vector expressing *gafY* and found it indeed restored GTA production, as with the single *rogA* deletion (see Figure below). This finding suggests that the deletion of *gafY* does not have a major polar effect on *gafZ* expression, as *gafZ* is necessary for GTA production and therefore must still be expressed in the double deletion. We did not feel this was a major point worth including in the paper, but can do so if the reviewer thinks it warrants inclusion.

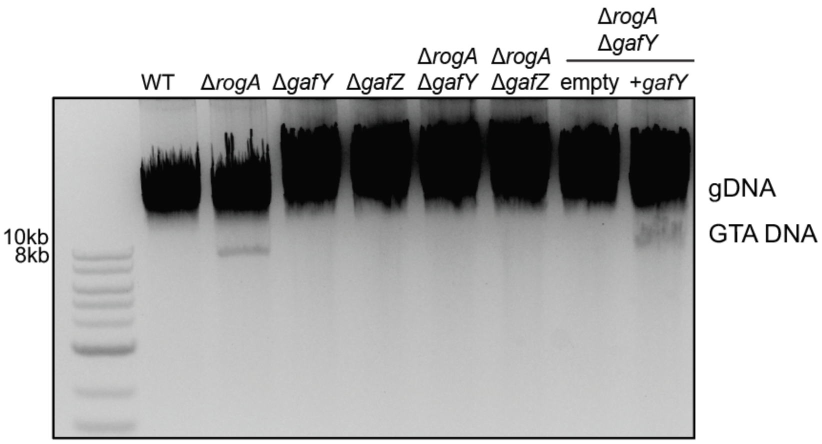

If I zoom way in on figure 2B (xylose) it looks like there might be a very faint GTA DNA band in the gafY lane?

Yes, we agree that there is a very faint band there. As noted in Fig. 2A, *gafZ* is required for GTA production. We think the likeliest explanation is that the ectopic GafY stimulates a bit of expression from the native *gafYZ* locus (our ChIP-seq data indicated that GafY binds its own promoter) and the resulting GafZ can combine with GafY to drive a small amount of GTA production. It seemed too speculative and not central to the paper to comment on this.

I am a bit confused about some of the ChIP-seq data. It seems the implication is that GafZ is only involved in transcription of the one locus, whereas GafY is involved in transcription of all

relevant loci. This strikes me as a bit off and could be investigated or discussed further. Presumably only GafY binds to the DNA whereas GafZ does not - is GafZ then captured by cross-linking to another protein that is bound to the DNA?

Indeed, we also find it intriguing that GafZ is, based on the ChIP-seq studies, only involved at the long, main GTA locus whereas GafY binds more broadly. Notably, GafZ also appears to locate in the ChIP studies downstream of GafY and the promoter. These observations suggest to us that GafZ may play a role in anti-termination of the long GTA locus. We felt it was too speculative and a bit off topic for this paper to comment on, but it is certainly something we intend to pursue in the future.

Another puzzle is the DNA transfer without tails. To my knowledge this is completely unprecedented for a "tailed phage" type of phage-like structure and there are tail genes expressed in the GTA cluster. Given this, it makes the DNase experiment mentioned previously even more relevant.

As noted above, we have now performed the experiment suggested and found that adding DNase does not impact transfer rates (Fig. 4E). Additionally, it is our understanding that some phages with very short tails also do not have visible tails in TEM.

It would be nice if the authors could provide some speculation on how the cells lyse to release the GTAs.

We have attempted to find homologs of the endolysin/holin genes involved in cell lysis in *R. capsulatus*, but were unsuccessful. Analysis of other genes upregulated during GTA expression in *C. crescentus* also did not reveal any putative endolysins/holins. As such, we are unable to speculate so far on what gene(s) drive cell lysis. We comment on this in the revised Results (lines 240-242).

It would also be nice to have some additional information on some of the additional loci that are upregulated with the GTA cluster - e.g., any clues based on homology what gene 1082 might encode?

We have now included additional information on the other loci that were upregulated (lines 165-170). Only *ccna_01082* had any predicted function based on homology (homeodomain-related), while the other genes were all hypothetical with no predicted function.

A few minor points:
line 139 has something off with the wording

We have adjusted the wording.

line 146 - at this point I feel like the DNA band is still putatively the GTA DNA?

We changed this to indicate it's putative GTA DNA.

line 408 - it is mentioned later, but since cell density is mentioned here it could be added about known quorum sensing effects on GTA production in Rhodobacter and Dinoroseobacter?

Yes - we now note the relationship of quorum sensing to GTA production in those other organisms at this point in the Discussion (line 464-465 in revised manuscript).

Reviewer #3: Gene transfer agents promote survival and DNA repair during stationary phase for Caulobacter crescentus by Gozzi et al.

In this manuscript, the Laub lab identified a GTA cluster in the bacterium Caulobacter crescentus. The authors successfully described this novel GTA and found its regulation mechanism, via a novel GTA regulator, the transcription factor RogA, that represses GafY and GafZ, homologs of GafA in R. capsulatus. They also found that GTA particles production gives a selective advantage to survive when cells reach stationary phase and when they suffer DNA damage. This work is an important contribution to the field of GTA. It also provides important insights into DNA transfer and cell survival in Caulobacter.

I have very few concerns about this manuscript. I think the data presented here are convincing, the experiments well-thought, and the conclusions solid. However, I still have a few suggestions that would improve the quality of the manuscript.

Major comments:

1. The authors suggest that " RogA indirectly regulates didA by somehow modulating DriD's expression or activity, possibly by causing an accumulation of double-stranded DNA breaks... " (lines 110-112) . This is an interesting point that deserves more attention. If RogA, DidA and DriD are in the same pathway:

a. Is RogA also regulated by DNA damage or is it upstream in the DriD/DidA regulation pathway? What is PdidA promoter activity in the presence of DNA damage in the strains tested in Fig 1C?

This is a great recommendation. We have now included an assay to test where *rogA* falls in the pathway (new Fig. S1). We measured the *didA* transcriptional reporter in WT, Δ*rogA*, and Δ*driD* cells with or without the DNA damaging agent zeocin. We observed no further induction of the *didA* reporter in the *rogA* mutant when DNA damage was added, suggesting that *rogA* is upstream of *didA* and that likely the reason the Δ*rogA* strain experiences an upregulation of *didA* expression is from a secondary effect of GTA packaging and widespread DNA damage.

b. DidD and DidA inhibit cell division (Fig. 1A), so how is cell division in the ΔrogA mutant? And what about in gafYZ-overexpression strains? From Fig. 4C, it seems like the Pxyl-gafZY strain looks fine, but is this consistent with the described DriD/DidA connection?

We have not observed any apparent delay in cell division in the *rogA* mutant in our hands. Given that production of GTAs appears to be lethal, it is hard to attribute any effects of cell-division inhibition to GTA activation, as those cells producing GTAs (and likely activating *didA*) will go on to lyse rather than just inhibit cell division. We have not observed any obvious cell-length differences in cells expressing *gafYZ* or cells bearing a *rogA* deletion, such as is seen in Fig. 4A,

as noted. However, the assays to look at cell division inhibition are almost always more sensitive in exponential phase when the cells are growing faster, whereas our assays are almost always during stationary phase, during which the role of cell-division inhibition is much less understood. In the future, if we can identify a gene needed for cell lysis, we could separate out lysis from GTA production and begin to test whether there is any evidence of cell division inhibition at stationary phase without the confounding variable of cell lysis.

c. Finally, how do ΔrogA cells cope with DNA damage in the DriD/DidA context? The last result section ("GTAs promote survival following DNA damage") shows a substantial increase in survival of the ΔrogA strain after DNA damages, and it seems to be entirely GTA-dependant. How does it connect back to DriD/DidA?

We thank the reviewer for this suggestion. We do not yet know the role of DriD and DidA in GTA production, but we have now tested the impact of DriD. We tested whether GTA-producer cells lacking *driD* can still provide a benefit to wild-type cells during stationary phase. We found that Δ*rogA* producers also lacking *driD* can still promote survival of the wild-type strain, equivalent to a Δ*rogA* strain, suggesting that DriD may not play a role in GTA production (see new Fig. S6A, lines 300-303). Although we identified *rogA* by studying the DriD pathway, it is possible that these pathways may not be directly linked; the production of GTAs likely leads to widespread DNA damage that will trigger DriD, independent of a direct connection or any relevance of the DriD pathway on GTA production.

2. Among the same lines, the authors clearly show here that a ΔrogA mutant and a gafYZ overexpressing strain have similar phenotypes, as RogA represses gafYZ expression. Is it a direct correlation between DidD/DidA, gafZY expression and GTA production? What is the PdidA promoter activity when gafY and gafZ are overexpressed?

This assay is technically somewhat challenging, as *gafYZ* overexpression is lethal to the cells and leads to widespread lysis, complicating the measurement of OD_{600} for cell material which is how the promoter activity data is normalized. We have included a β-galactosidase assay now in the supplemental (Fig. S2D) of *gafYZ* expression when cells enter stationary phase, akin to conditions used for inducing GTA expression. We observed about a ~4-fold increase in *didA* expression, similar to the normalized upregulation observed in the *rogA* mutation in Fig. 1C.

We have included below plates showing cells harboring the *didA* reporter grown with and without X-gal and bearing either an empty vector or a construct for overexpressing *gafY* alone or *gafYZ* together. The cells expressing *gafYZ* cannot grow under constitutive expression, so it is impossible to judge their induction of the *didA* promoter. We do, however, see strong activation of the *didA* promoter with just the overexpression of *gafY*.

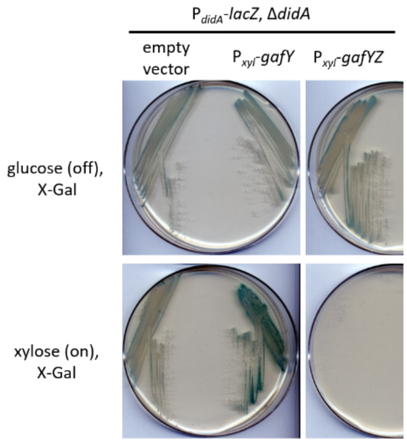

3. It has been shown previously that, in R. capsulatus, GafA activity and GTA are regulated by CtrA. CtrA has been extensively studied in Caulobacter. Is this master regulator involved in GTA regulation in Caulobacter? In the discussionm, the authors state that "... the CckA-ChpT-CtrA phosphorelay [...] regulate(s) GTA synthesis in Rhodobacter (30-34), but not in Caulobacter." (lines 448-450), but they need to provide more context / data to back-up this claim for the non-Caulobacter expert readers.

We now cite Laub et al 2002, which mapped the CtrA regulon in *Caulobacter* through global gene expression profiling and ChIP-seq and did not find any evidence for regulation of *gafYZ* or the GTA loci.

4. The authors showed here that Caulobacter GTA are mostly produced in stationary phase. What is the mechanism? In other species, GTA can be regulated by ppGpp and/or nutrient starvation. Is it the case in Caulobacter? All experiments performed in the present work were done using rich PYE medium. What happens to GTA production when Caulobacter cells are starved? What about in a ΔspoT strain that cannot produce ppGpp?

The precise mechanism or signal in stationary phase that triggers GTA production in *Caulobacter* is not yet clear. We have now deleted *spoT* in cells also lacking *rogA* and assayed cells for GTA production via Western blot for a GTA protein (panel A below) and DNA extraction (panel B), as well as examining cell viability following GTA induction (panel C). We find that cells lacking *spoT* (and lacking *rogA*) produce less GTA protein, produce less GTA DNA, and exhibit less cell death - these results suggest that ppGpp may promote GTA biogenesis, but precisely what starvation signal triggers SpoT is not yet clear so we have chosen to leave the data below out of the manuscript.

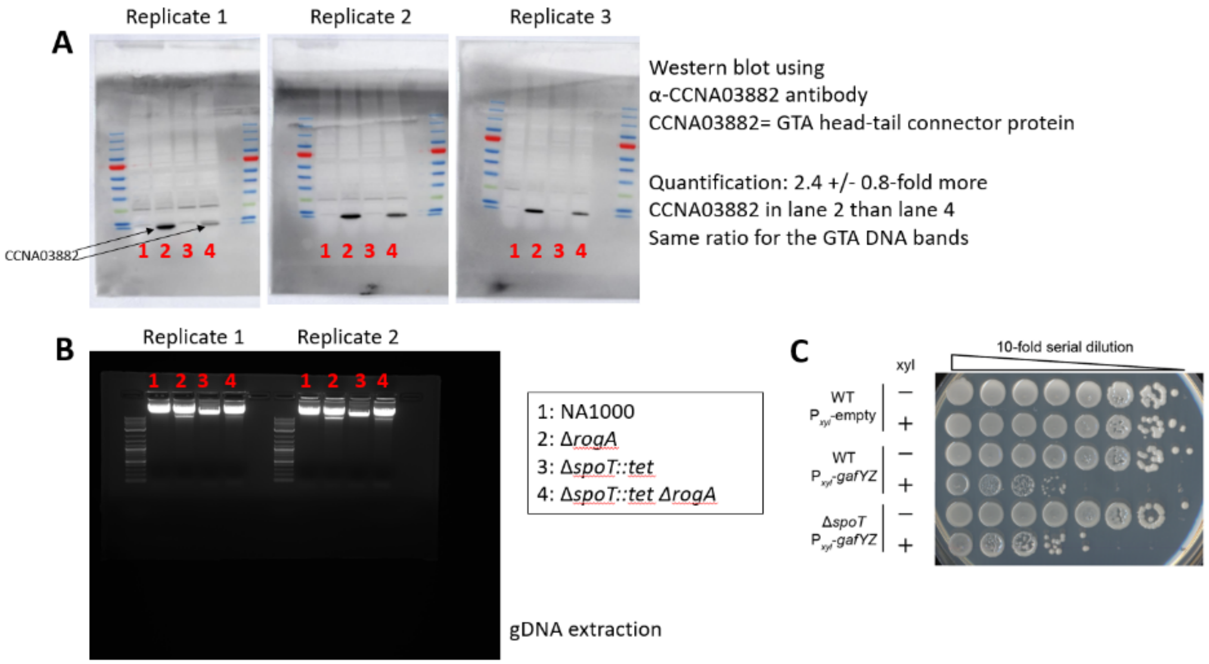

5. Fig. 3B clearly shows that overexpressing gafY and gafZ increases cell death. The authors then provide quantification for "lysis" (measured as released protein concentration as a proxy) in Fig. 3C. The experiments are done in PYE, which is mainly made of peptone and yeast extract. How accurate measurement can be provided using this medium (the method section lacks information, see minor comments)? Furthermore, as overexpressing gafYZ rises the number of GTA produced, how can the authors conclude that the increase in extracellular protein content they detect is solely linked to lysis, and not GTA particle release in other way? It seems like other techniques to assay lysis would be more appropriate (such as enzymatic assays of cytoplasmic proteins in cell-free supernatants, western blot using a known cytoplasmic protein, or live/dead staining, for example).

We have now included a time course (Fig. S5D) staining for dead cells as we overexpress *gafYZ,* with dead cells beginning to appear after 2 hours of induction, in line with the cell lysis data in Fig. 3B, and with most cells staining dead after 4 hours. Although these measurements are done in PYE, we are, of course, measuring the increase in released protein in Fig. 3C (and 3D), so any contributions from the peptone and yeast extract to the signal are effectively normalized out. Additional information on the method used have been added to the Methods section (lines 778-781).

6. What is the cell viability of a ΔrogA mutant over time compared to WT? Fig. 3D only provides extracellular protein content and cell density. Released protein content spikes in the ΔrogA mutant, while cell density is similar for both WT and ΔrogA. Can the author comment on that? What are the CFUs data for both strains under those conditions?

We thank the reviewer for this suggestion. We now have performed a timecourse of WT and Δ*rogA* cultures starting at OD_{600} = 0.75 and taking samples every 45 minutes for CFU plating

(see new Fig. S5C). Even though the apparent OD_{600} is approximately equal between the strains, as is seen in Fig. 3D, we observed a significant decrease in CFU in the Δ*rogA* strain relative to WT after about 3 hrs that persisted throughout the remainder of the time course, suggesting there is significant loss of cell viability during GTA production in the *rogA* mutant.

7. Fig 4 A-B: It would be interesting to see the results for the SYBR-gold staining for the ΔrogA mutant and the TEM for the supernatant of the gafYZ overexpressing strain (especially as the authors used both strains as "GTA-donors" later on (see point #10)).

We now include TEM from the PEG-precipitated supernatant of the *gafYZ* overexpression strain in Fig. S5G.

8. "No visible tails" (line 232) could be detected in the TEM images (Fig. 4B), while the genes encoding the tail proteins are present in the GTA encoding locus (Fig.1D-E). Can the author comment on that?

We have added a clarification (lines 253-255) that the tail may be too short to resolve and further emphasized that indeed we do see tail genes in the cluster.

9. The authors nicely show GTA-mediated DNA transfer from one Caulobacter crescentus CB15N to another CB15N cell. What about DNA transfer to other Caulobacter strains and closely related Caulobacterales species?

This is beyond the scope of this work, but something we intend to study in the future. We have added a comment in the Discussion highlighting this fact and that future studies may help to reveal any species specificity of the *Caulobacter* GTA.

10. Fig. 5 and 6: Why are "GTA-producing donors" different in the all the co-cultures experiments. Is there a rationale to use ΔrogA strains in some experiments and gafYZ overexpressing strains in other?

We have now added a clarification (lines 284-287) regarding the difficulty of using *gafYZ* overexpression strains for long-term co-incubations.

Minor comments:

1. The method section sometimes lacks key information (please read all and add relevant details when needed, important for the non-Caulobacter experts). For example:
a. The authors worked with a marked rogA mutant (ΔrogA::tetR), while clean in-frame unmarked deletions are commonly used in C. crescentus. Please, briefly explain the rationale for not choosing to do a unmarked deletion.

We have added a comment in the Methods (line 819-820) to address why the marked *rogA* deletion was useful in co-incubation experiments for quantifying donor and recipient strains afterward. This marked deletion was also useful for simplifying strain construction using transduction rather than recreating in-frame clean deletions for each new strain.

b. I missed the method used for the quantification of released protein content as a proxy for cell lysis.

We have added a clarification of the method for released protein content quantification in the Methods (lines 778-781).

c. The authors chose to integrate PdidA-lacZ, Pxyl-gafYZ and other markers to the hfaB locus in the genome. Can the authors briefly explain their choice.

We have added a clarification of the common use of the dispensable *hfaB* locus (line 654-656).

d. The method used to quantify each population in co-cultures (used to generate the data shown in Fig.5) is unclear and lacks details.

We now provide further explanation in the Methods about how each population was quantified in the experiments shown in Fig. 5D. To aid in explanation of 5C, we have clarified the genetic background of the WT and Δ*rogA* strains in the figure, and emphasize in the figure caption that cells were plated on plates containing the respective antibiotics (lines 1285-1287).

e. I missed the methods for UV damage induction (exposure time?).

We now indicate the exposure amount and times in the Methods (lines 832-834).

2. Lines 138 - 139: " total DNA was isolated and separated by gel electrophoresis from cells in stationary phase, which was when we didA expression was highest in the ΔrogA mutant". Please rephrase.

This sentence was rephrased for clarity.

3. Fig. 3E: It would be nice to see the total plot, with the relevant log2 from 2 to 15 portion as an insert.

We now present the total plot in Fig. 2E, with the relevant portion highlighted with color-coded points.

4. Fig 4E: Please provide the number of replicates done in the legend and add error bars to the graphs (as it is done for all other experiments).

We have now noted the number of replicates in the legend to Fig. 4E and added error bars to graphs throughout the manuscript.

5. I am assuming that data illustrated in Fig. 5C and 5D are generated in a similar way (the manuscript is lacking information about this, see minor point 1d above). For a better readability, I would suggest for both experiments to be presented the same way.

Thank for this note, we have now included further explanation in the methods for how these data was generated (lines 820-830). In general, the data were generated in a similar way, except that for the markerless *rec-* strain, colonies were enumerated by replica plating and counting cells that were not the resistance of the donor strain. This allowed us to quantify the *rec-* strain, as

opposed to just using an antibiotic selection for enumeration as was done earlier in Fig. 5. Therefore, it was only possible to display the final counted data as ratios rather than the replica plates.

6. Same comment for Fig. 5E and 5F.

For Fig. 5E and 5F, the relative survival (treated/untreated) was first relevant for each individual strain on its own during UV treatment. However, in Fig. 5F, we chose to show the raw images so that the data was not compressed down to comparing ratios with different supernatants, seeing as the conclusions drawn were across samples, whereas for Fig. 5E the ratios determined were comparing the same strain to itself before and after treatment.

7. Fig. 6A: the chloramphenicol resistance cassette should be depicted in the scheme describing the experiment.

Thanks for the suggestion - this has been added.

8. Fig. 6D: Is the difference in survival against EMS significant? Providing relevant statistics here might be a nice addition.

We now provide statistical analysis of the data, which indicates that the survival against EMS, though modest in effect size is statistically significant.
