# Supplementary material for: Prophage-like gene transfer agents promote Caulobacter crescentus survival and DNA repair during stationary phase
Source: PLoS Biol. 2022 Nov 3;20(11):e3001790. doi: 10.1371/journal.pbio.3001790 (PMC9632790; doi:10.1371/journal.pbio.3001790)
Supplement: Attachment — Submitted filename: GTA_paper_reviews D05.pdf [file pbio.3001790.s014.pdf]

We thank the reviewers for their additional comments and responses to our revisions. Below we respond to both comments and indicate changes we have made accordingly. Reviewer text is in black, our responses are in blue.

Reviewer #1: I thank the authors for having tackled my comments and criticisms seriously. I appreciate the novel analyses, statistical tests and discussion. I've no further objections or criticisms. As a note to the authors response (this is without impact to the evaluation of the paper): "Perhaps most notable is the fact that cells acquiring DNA by transformation cannot 'know' the origin of the incoming DNA and whether it is conspecific or not. " This statement is incorrect. In Proteobacteria several systems exist of uptake signal sequences allowing just that. In Firmicutes this recognition can be achieved by pheromones. So I do think there are extensive parallels between acquiring DNA by transformation and GTA. But I'm fine with the current level of the discussion.

We recognize this is a comment just regarding a statement in our previous "Response to Reviewers" rather than a comment on text in the manuscript. However, we have added in an extra discussion of this point to draw a connection between GTAs and uptake sequences in transformation in our manuscript (lines 491-493).

Reviewer #2: The authors have thoroughly responded to the comments made during the initial reviews. I am only left unsatisfied with one point, which is related to the structure of the GTA particles and explanation given for the lack of tails. The authors state that it is possible there are tails there but that they are too short to observe in the TEM images. Is the implication that these GTAs are now akin to podophage structures? I.e., as opposed to siphophage like their relatives in other species (Rhodobacter and Dinoroseobacter)? The C. crescentus GTA cluster contains some of the tail gene homologs, but not all - is it proposed that this reduced gene set allows gene transfer, but in the absence of an extended tail structure? Considering the high resolution structure that is now available for RcGTA (Bardy et al 2020), how do we reconcile the potential tail protein functions present an absent and how DNA might be transferred? This is where information about which of these genes are required or not for the GTA activity to occur, but I do not want to suggest this as a condition for this manuscript - however, it seems to warrant more discussion at least and some mention of potential future steps to address this. It actually has some fairly broad implications if these particles can function for DNA transfer with such seemingly incomplete tails. I believe it is also the case that some TEM preparation/staining techniques can cause some phage particles to lose their tails so perhaps this should be investigated and discussed?

Thank you for this comment. The result was surprising to us as well and we have now addressed this discrepancy with the *Rhodobacter* GTA structure throughout the text. We have added additional discussion of the lack of tails (including referencing which tail genes are in common between *Caulobacter* and *Rhodobacter* and suggestion of a potential methodological cause of tail loss) in the Results (lines 256-259). We have also included further comments in the Discussion and suggestions of experiments to perform in the future (lines 495-499) to help resolve this discrepancy.
